# Navigation in bile acid chemical space: discovery of novel FXR and GPBAR1 ligands

**DOI:** 10.1038/srep29320

**Published:** 2016-07-06

**Authors:** Claudia Finamore, Carmen Festa, Barbara Renga, Valentina Sepe, Adriana Carino, Dario Masullo, Michele Biagioli, Silvia Marchianò, Angela Capolupo, Maria Chiara Monti, Stefano Fiorucci, Angela Zampella

**Affiliations:** 1 Department of Pharmacy, University of Naples “Federico II”, Via D. Montesano, 49, 80131 Naples, Italy; 2 Department of Surgery and Biomedical Sciences, Nuova Facoltà di Medicina, P.zza L. Severi 1, 06132 Perugia, Italy; 3 Department of Pharmacy, University of Salerno, Via Giovanni Paolo II, 132, 84084 Fisciano (Salerno), Italy

## Abstract

Bile acids are signaling molecules interacting with nuclear receptors and membrane G-protein-coupled receptors. Among these receptors, the farnesoid X receptor (FXR) and the membrane G-coupled receptor (GPBAR1) have gained increasing consideration as druggable receptors and their exogenous dual regulation represents an attractive strategy in the treatment of enterohepatic and metabolic disorders. However, the therapeutic use of dual modulators could be associated to severe side effects and therefore the discovery of selective GPBAR1 and FXR agonists is an essential step in the medicinal chemistry optimization of bile acid scaffold. In this study, a new series of 6-ethylcholane derivatives modified on the tetracyclic core and on the side chain has been designed and synthesized and their *in vitro* activities on FXR and GPBAR1 were assayed. This speculation resulted in the identification of compound **7** as a potent and selective GPBAR1 agonist and of several derivatives showing potent dual agonistic activity.

Next to their ancestral roles in lipid digestion and solubilization, bile acids (BAs), the principal constituent of bile, are today recognized signaling molecules involved in many physiological functions and these signaling pathways involve the activation of several metabolic nuclear receptors, mainly the BAs sensor FXR^1^,^2^, and the dedicated membrane G-protein-coupled receptor, GPBAR1 (TGR5)^3^.

Principally, FXR functions as a sensor of bile acid level playing an important role in the regulation of their intracellular levels in hepatocytes^4^. FXR is activated by CDCA (**1**)^1^,^2^,^5^ and upon CDCA binding, FXR forms a heterodimer with the retinoid X receptor (RXR) that binds specific DNA sequences within the promoter regions of target genes. The canonical gene expression program activated by FXR leads to the reduction in the intracellular bile acid levels by increasing the export of bile acids out of cells, decreasing bile acid uptake and decreasing bile acid synthesis^6^,^7^,^8^. As a consequence, FXR has been identified as an appealing target in the treatment of cholestasis disorders such as primary biliary cirrhosis (PBC) and liver steatosis^9^,^10^,^11^, two severe human conditions in which bile acids homeostasis is impaired.

PBC is an immunologically mediated progressive liver disease characterized by the destruction of small intrahepatic bile ducts, with accumulation of bile acids in the liver and consequently inflammation, fibrosis, and potential cirrhosis. Fatigue and pruritus are the most common symptoms of primary biliary cirrhosis, and both can be debilitating in some patients. Cholestasis causes intense, sometimes intolerable, itch leading to scratching, excoriation, sleep deprivation, and depression^12^.

In addition, FXR plays a crucial beneficial role in hepatic triglyceride homeostasis, as well as in glucose metabolism and therefore, FXR agonists are also promising for the treatment of non-alcoholic fatty liver disease (NASH), dyslipidemia and type 2 diabetes (T2DM)^13^,^14^,^15^,^16^.

In addition to FXR and other nuclear hormone receptors, BAs can also signal through a membrane- receptor (GPBAR1/TGR5/M-BAR)^3^. The most potent endogenous GPBAR1 activator is TLCA (**2**) followed by DCA, while other BAs are less potent. GPBAR1^−/−^ mice display prolonged cholestasis, exacerbated inflammatory response and more severe liver injury after partial hepatectomy^17^. In addition, in a mouse model of xenobiotic (DDC)-induced sclerosing cholangitis, mice overexpressing GPBAR1 showed less liver injury while mice lacking GPBAR1 showed aggravation of inflammation and fibrosis^18^. Collectively, these findings suggest a critical role of GPBAR1 for liver protection against BA overload but activation of GPBAR1 should be also associated with severe side effects, especially in the context of impaired bile acid level in the liver.

In fact, GPBAR1 has been recently identified as the physiological mediator of pruritus^19^, a common symptom observed in cholestasis and the severity of this side effect limits the pharmacological utility of dual agonists in the treatment of primary biliary cirrhosis (PBC) and related cholestatic disorders.

On the other hand, responses to GPBAR1 activation include increased energy expenditure, improved intestinal motility, glucose metabolism and insulin sensitivity^20^,^21^. The latter two occur through the release of the glucagon-like peptide 1 (GLP-1) by intestinal L cells upon GPBAR1 activation^22^. Therefore, the exogenous regulation of this receptor represents an attractive strategy to treat metabolic disorders such as NASH, hypercholesterolemia, hypertriglyceridemia, and T2DM^23^,^24^. Thus, in the window of metabolic disorders, the development of ligands endowed with dual activity toward GPBAR1 and FXR appears to be a promising strategy^23^,^24^,^25^,^26^. In contrast, the discovery of highly selective FXR agonists could represent a new frontier in the treatment of primary biliary cirrhosis (PBC) and related cholestatic disorders where the concomitant activation of GPBAR1 could increase patient risk for adverse side effects. Indeed, results from PBC phase II clinical trial with 6-ECDCA/OCA (**3**), a potent semi-synthetic steroidal FXR agonist^27^, have shown that while the compound exerts benefit, its use has associated with pruritus. In fact, up to 40% of PBC patients halted the treatment due to the severity of itching in one trial and approx. 80% of patients experienced the symptoms. The reason why OCA causes itching is unclear. However, this agent is almost equally potent on FXR and GPBAR1 28,29 and it is predictable that the severity of this side effect could prevent its use in stage III and IV PBC patients^30^.

Medicinal chemistry on 6-ECDCA scaffold and on bile acid scaffold has produced several derivatives modified on the side chain in the length and in the nature of the end-group and on the tetracyclic core^11^,^31^,^32^,^33^. Indeed these derivatives cover the same chemical space of BAs that are intrinsically promiscuous toward FXR and GPBAR1 and therefore, with few exceptions, this kind of speculation mainly afforded dual modulators^33^. The most interesting results have been obtained with compounds **4–6** (Fig. 1).

The replacement of the negative charged end group with a neutral polar group produced derivative **4**, again a potent dual agonist and the above activity was also maintained by the corresponding *nor*-derivative **5**, with one carbon less on the side chain. Of interest compound **5** attenuates liver damage in animal models of non-obstructive cholestasis without inducing itching^34^. Finally, speculation on stereochemical modification on ring B produced **6**, a C-24 alcohol that represents the first example of ursodeoxycholane derivative substituted at C-6 with a β-oriented ethyl group. Pharmacological assays demonstrated that this derivative is a rather potent ligand for GPBAR1 (EC_50_ 1.03 μM) failing in transactivating FXR at any concentration tested^26^.

Recently we have also demonstrated that compound **6** exerts portal pressure-lowering effects in rodent models of portal hypertension by directly regulating the expression/activity of cystathionine γ-lyase (CSE) and endothelial nitric oxide synthases (eNOS) in liver sinusoidal endothelial cells (LSEC), thus affirming this compound as a novel approach to attenuate the hemodynamic changes in patients with liver cirrhosis^35^.

In addition, in an our recent contribution, we have extended the structure-activity relationship on C24 6-ethylcholane scaffold modifying the hydroxyl group at C-3 and demonstrating that the elimination or the inversion of the above functionality on ring A could shift the activity toward FXR^36^.

Prompted by these promising results, we decided to expand our investigation proceeding in the modification of the side chain length on the 6-ethylcholane scaffold. As shown in the Fig. 2, a small library of C23 6-substituted cholane derivatives, compounds **7–15**, have been prepared.

In this framework, the effects of the stereochemical arrangement of the cholane C-6/C-7 positions and the substituents adorning the C-3 position and the C-23 side chain end group (OH or COOH) have been explored in term of potency/selectivity toward FXR and GPBAR1.

In addition, the chemical diversity of available bile acid receptor modulators have been increased preparing bis-*homo* 6-ethylcholane derivatives **16–19**. Pharmacological investigations resulted in the identification of compounds **7** and **19** as a potent and selective GPBAR1 agonist and a potent dual agonist respectively.

## Results

**Figure 3:**
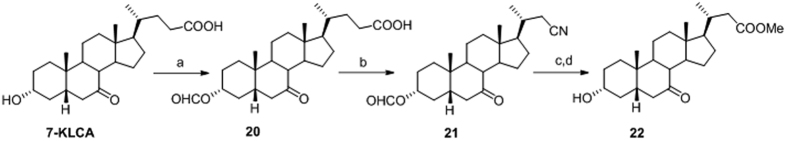
Preparation of 7-keto-norLCA methyl ester (22). *Reagents and conditions*: (a) HCOOH, HClO_4_; (b) TFA, trifluoroacetic anhydride, NaNO_2_; (c) KOH 30% in MeOH/H_2_O 1:1 v/v, 66% over three steps; (d) p-TsOH, MeOH dry, quantitative yield.

In the synthesis of 6-ethyl *nor*cholane derivatives **7–15**, the first step was the preparation of the key intermediate 7-keto-*nor*LCA methyl ester **22** from 7-KLCA (Fig. 3). A reaction sequence comprising Fisher’s esterification with formic acid and perchloric acid generated the formiate derivative **20** that was subjected to a Beckmann rearrangement by treatment with sodium nitrite in a trifluoroacetic anhydride/trifluoroacetic acid mixture obtaining the 23-nitrile derivative **21**^37^. Prolonged alkaline hydrolysis afforded the corresponding carboxylic acid that was in turn transformed in the methyl ester derivative **22** by methanol/p-toluensulfonic acid treatment (66% yield from 7-KLCA).

### Preparation of 3α-hydroxy-6-ethyl*nor*cholane derivatives

**Figure 4:**
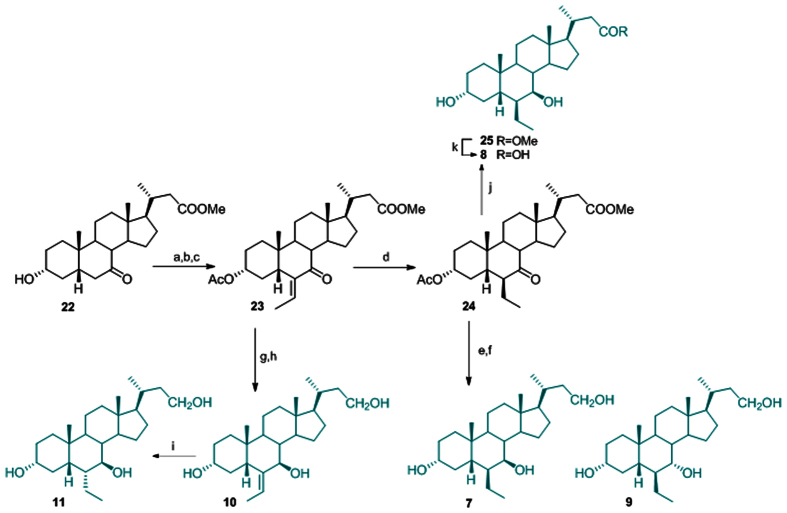
Synthesis of 3α-hydroxy-6-ethyl*nor*cholane derivatives. *Reagents and conditions*: (a) acetic anhydride, pyridine; (b) DIPA, *n*-BuLi, TMSCl, TEA dry, THF dry −78 °C; (c) acetaldehyde, BF_3_(OEt)_2_, CH_2_Cl_2_, −60 °C, 60% over three steps; (d) H_2_, Pd(OH)_2_, THF/MeOH 1:1, quantitative yield; (e) NaBH_4_, MeOH dry, 0 °C; (f) LiBH_4_, MeOH, THF dry, 0 °C, 77% over two steps; (g) NaBH_4_, MeOH; (h) LiBH_4_, MeOH dry, THF, 0 °C, 85% over two steps; (i) H_2_, Pd(OH)_2_, THF:MeOH 1:1 v/v, quantitative yiel; (j) NaBH_4_, MeOH dry, 0 °C; (k) NaOH, MeOH:H_2_O 1:1 v/v, 82% over two steps.

Acetylation at C-3 on **22** and aldolic addition to a silyl enol ether intermediate generated the intermediate **23** (60% over three steps) that was hydrogenated at the exocyclic double bond (H_2_ on Pd(OH)_2_) affording exclusively the 6β-ethyl group in the compound **24** (quantitative yield). Treatment of **24** with an excess of NaBH_4_ in methanol followed by LiBH_4_ reduction on the crude reaction product to secure the reduction at the methyl ester on the side chain, gave the concomitant deacetylation at C-3 and reduction at C-7 keto and at C-23 methyl ester groups (Fig. 4).

HPLC purification gave the main product **7** and small amount of its epimer at C-7, compound **9**. When intermediate **24** was treated with a stoichiometric amount of NaBH_4_, the reduction occurred exclusively at C-7 keto group giving the methyl ester **25**, that was subjected to basic hydrolysis furnishing the carboxylic acid **8** in 82% over two steps.

Intermediate **23** was also used as starting material in the preparation of **10** and **11**. Sodium borohydride/LiBH_4_ treatment on **23** proceeded in a stereoselective manner, affording the exclusive formation of 7β-hydroxyl derivative as judged by the shape of H-7 as a doublet (*J* = 9.8 Hz) which is consistent with an axial disposition for this proton, and therefore with the β-orientation of the hydroxyl group on ring B. Dipolar couplings H-7/H-24 and Me-25/H-5 in the NOESY spectrum allowed definition the E configuration for the exocyclic double bond as depicted in **10**. Hydrogenation on Pd(OH)_2_ catalyst afforded derivative **11** with the ethyl group at C-6 α-oriented.

### Preparation of 3-deoxy- and 3β-hydroxy-6α-ethyl*nor*cholane derivatives

**Figure 5:**
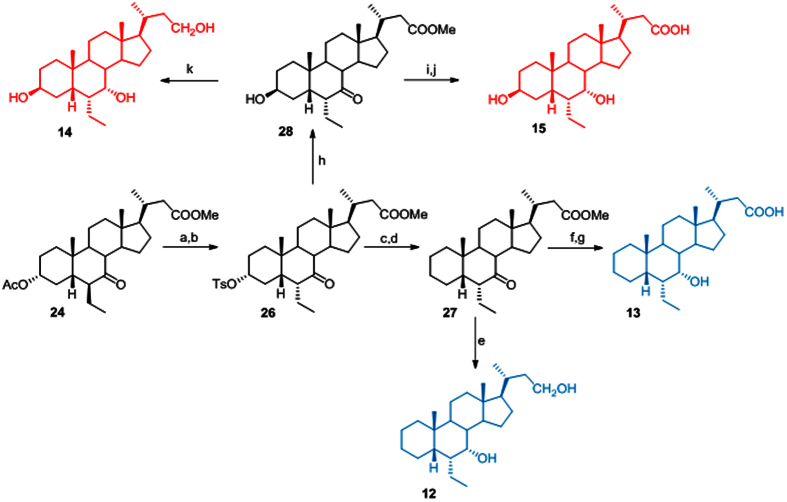
Synthesis of 3-deoxy- and 3β-hydroxy-6α-ethyl*nor*cholane derivatives. *Reagents and conditions*: (a) MeONa, MeOH; (b) p-TsCl, pyridine, 73% over two steps; (c) LiBr, Li_2_CO_3_, DMF, reflux, (d) H_2_, Pd(OH)_2_, THF/MeOH 1:1, room temperature, quantitative yield over two steps; (e) LiBH_4_, MeOH dry, THF, 0 °C, 70%; (f) NaOH, MeOH:H_2_O 1:1 v/v; (g) LiBH_4_, MeOH dry, THF, 0 °C, 92% over two steps; (h) CH_3_COOK, DMF:H_2_O 5:1 v/v; (**i**) NaOH, MeOH:H_2_O 1:1 v/v; (j) LiBH_4_, MeOH dry, THF, 0 °C, 58% over two steps; (k) LiBH_4_, MeOH dry, THF, 0 °C, 57%.

In the preparation of 3-deoxy- and 3β-hydroxy-6-ethyl*nor*cholane derivatives **12–15**, a convergent protocol was applied starting from the methyl ester **24**, that was first treated with MeONa in methanol to effect de-acetylation at C-3 and inversion at C-6 and then with tosyl chloride to afford **26** in 73% yield over two steps (Fig. 5).

Elimination by LiBr/Li_2_CO_3_ treatment and hydrogenation of the unsaturated-ring A transient intermediate furnished **27** that was used as starting material for the preparation of compounds **12** and **13**.

LiBH_4_ treatment produced the concomitant reduction at C-24 methyl ester and at C-7 carbonyl group furnishing **12** whereas alkaline hydrolysis of the methyl ester followed by LiBH_4_ treatment gave **10** in high chemical yield.

Finally, treatment of the tosyl derivative **26** with potassium acetate in DMF/H_2_O afforded inversion at C-3 on **28**. Reduction at C-7 and C-24 with LiBH_4_ and hydrolysis at methyl ester group on the side chain gave **14** and **15**, respectively.

### Preparation of bis-*homo* 6-ethylcholane derivatives

**Figure 6:**
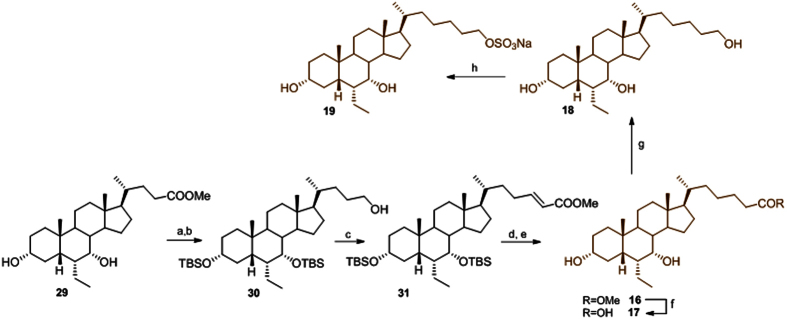
Preparation of bis-*homo* 6-ethylcholane derivatives. *Reagents and conditions*: (a) 2,6-lutidine, t-butyldimethylsilyl trifluoromethanesulfonate, CH_2_Cl_2_, 0 °C; (b) LiBH_4_, MeOH dry, THF, 0 °C, 68% over two steps; (c) DMSO, oxalyl chloride, TEA dry, CH_2_Cl_2_, −78 °C then methyl(triphenylphosphoranylidene)acetate, 79%; (d) H_2_, Pd(OH)_2_/C Degussa type, THF/MeOH 1:1, quantitative yield; (e) HCl 37%, MeOH, 88%; (f) NaOH 5% in MeOH/H_2_O 1:1 v/v, 89%; (g) LiBH_4_, MeOH dry, THF, 0 °C, 78%; (h) Et_3_N.SO_3_, DMF, 95 °C, 25%.

In the preparation of *bis*-homo-6-ethylcholane derivatives **16**, a four-steps reaction sequence on **29**, previously prepared in our laboratory^25^,^26^, including protection of the alcoholic functions at C3 and C7, reduction of the side chain methyl ester, and subsequent one pot Swern oxidation/Wittig C2 homologation, gave the protected methyl ester of Δ^24^,^25^ bis-*homo*ECDCA **31** (Fig. 6).

Side chain double bond hydrogenation and alcoholic function deprotection gave the methyl ester **16** that was used as starting material in the preparation of the carboxylic acid **17** and the corresponding alcohol **18** through treatment with LiOH and LiBH_4_, respectively. Chemoselective sulfation at C-26 hydroxyl group on a small aliquot of **18** gave the corresponding sulfate derivative **19** 38.

## Discussion and Conclusion

**Figure 7:**
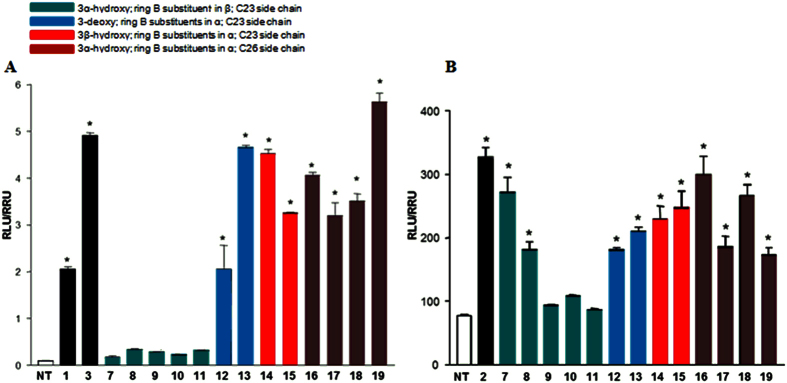
Agonism on bile acid receptors by transactivation assay. (**A**) HepG2 cells were transfected with pSG5-FXR, pSG5-RXR, pCMV-βgal, and p(hsp27)TKLUC vectors. Cells were stimulated with compounds **7–19** (10 μM). CDCA (**1**, 10 μM) and 6-ECDCA (**3**, 1 μM) were used as a positive control. (**B**) HEK-293T cells were co-transfected with GPBAR1 and a reporter gene containing a cAMP responsive element in front of the luciferase gene. Cells were stimulated with **7–19** (10 μM). TLCA (**2**, 10 μM) was used as a positive control. Luciferase activity served as a measure of the rise in intracellular cAMP following activation of GPBAR1. In both panels, results are expressed as mean ± standard error. *p < 0.05 *versus* not treated cells (NT).

Derivatives **7–19** were tested in the luciferase reporter assays on HepG2 and HEK-293T cells transfected with FXR and GPBAR1, respectively. Data shown in Fig. 7, panel A, reporting the results of the transactivation assay on FXR, reaffirm the 6α/7α stereochemical arrangement around ring B as the most important feature in FXR activity with derivatives **7–11**, with one or two substituents on ring B in β configuration, devoid of any activity at 10 μM dose. Of interest is the behavior of derivatives **12–15**, with both substituents on ring B in α-configuration and a shortened (C23) side chain. In a cross comparison between the above compounds, it is quite evident that the presence of a negative charge on the side chain favors the 3-deoxy derivatives (compare **13** with COOH vs **12** with CH_2_OH), whereas the alcoholic function at C-24 improves FXR activity of the corresponding 3β-hydroxyl derivative (compare **14** with CH_2_OH vs **15** with COOH). In addition, results on derivatives **16–19** demonstrate that side chain elongation on the 6-ethyl scaffold could be instrumental in generation potent FXR agonists. In this subset, the nature of the side chain end group produces a remarkable effect in FXR transactivation with the sulfate derivative **19**, the most potent FXR agonist generated in this study.

Results of transactivations of CREB-responsive elements in HEK-293T transiently transfected with the membrane bile acid receptor GPBAR1 are showed in Fig. 7, panel B. Compounds **7** and **8**, with both the ethyl and the hydroxyl groups on ring B β-oriented, were demonstrated inducers of cAMP-luciferase reporter gene, with **7** showing a potency similar to that of TLCA (**2**), the most potent endogenous GPBAR1 agonist. As expected, all derivatives with both substituents on ring B in α-configuration are endowed with GPBAR1 agonistic activity, and the above activity is quite independent of the length and the functionalization of the side chain and by the substitution at C-3 with 3α-, 3β-hydroxy and 3-deoxy derivatives sharing a similar behavior. Even if, by comparing FXR and GPBAR1 transactivation results, derivatives **12–19** are to be considered dual agonists, there is a considerable difference in GPBAR1 activity of C26 derivatives **16–19**. It is quite evident that the presence of a neutral (COOMe in **16**) or a non-charged polar group (CH_2_OH in **18**) on the elongated side chain is preferable respect to a negative charged end group such as the COOH in **17** and the sulfate in **19**.

The relative potency of selected members of this novel family was then investigated by a detailed measurement of the concentration-response curve of the 3-deoxy C-23 carboxylic acid derivative **13**, the 3β-hydroxyl C-23 alcohol **14** and the C-26 sulfate derivative **19**, all sharing the 6α/7α configuration, on FXR and GPBAR1 transactivation.

**Figure 8:**
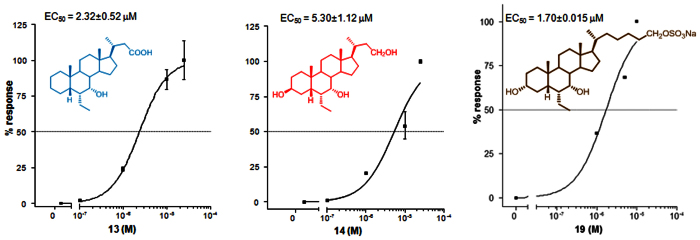
Concentration-response curve of 13, 14, and 19 on FXR. HepG2 cells were transfected with FXR as described above and used in a luciferase reporter assay. Twenty-four hour post transfection cells were stimulated with increasing concentrations of each agent: range from 100 nM to 25 μM. Results are expressed as mean  ± standard error.

**Figure 9:**
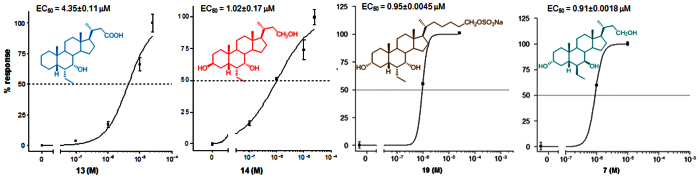
Concentration-response curve of compounds 13, 14 and 19 on FXR. HepG2 cells were transfected with FXR as described above and used in a luciferase reporter assay. Twenty-four hour post transfection cells were stimulated with increasing concentrations of each agent: range from 100 nM to 25 μM. Results are expressed as mean ± standard error.

As illustrated in Figs 8 and 9, compounds **13**, **14**, and **19** transactivate FXR with an EC_50_ of 2.3 μM, 5.3 μM and 1.7 μM, respectively. In addition, **13**, **14**, and **19** transactivate GPBAR1 with EC_50_ of 4.3 μM, 1.0 μM and 0.95 μM, respectively. Combining these data, compound **19** represents the most potent FXR/GPBAR1 dual agonist identified in this study. Finally, compound **7** exerted a concentration-dependent effect on activation of cAMP responsive element in HEK-293T cells transfected with GPBAR1 with an EC_50_ of 0.91 μM.

**Figure 10:**
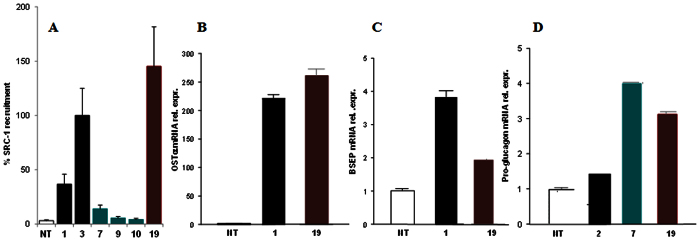
(**A**) Coactivator recruitment assay measuring a direct interaction of FXR with SRC-1; ligands at 2 μM. Results are expressed as percentage of the effect of **3** arbitrarily settled as 100%. NT is referred to the experiment in absence of ligand. Results are expressed as mean ± standard error. (**B,C)** Real-time PCR analysis of mRNA expression on FXR target genes BSEP (**B**), and OSTα (**C**) in HepG2 cells primed with 10 μM of compound **19**. CDCA (**1**) was used as a positive control at 10 μM. (**D)** Real-time PCR analysis of mRNA expression of GPBAR1 target gene Pro-glucagon in GLUTAg cells stimulated with 10 μM of compounds **7** and **19**, and TLCA (**2**) used as a positive control at 10 μM. Values are normalized to GAPDH and are expressed relative to those of not treated cells (NT) which are arbitrarily settled to 1. The relative mRNA expression is expressed as 2(−ΔΔCt).

The ability of compound **19**, the most potent FXR agonist in these series, in the recruitment of the coactivator SRC-1 was also measured through Alpha screen technology. CDCA (**1**) and 6-ECDCA (**3**) were used as positive controls at 2 μM concentration and the effect of 6-ECDCA (**3**) was settled as 100%. As shown in Fig. 10, panel A, compound **19** showed an activity in the recruitment of SRC-1 co-activator at least comparable, if not better, to that measured for **3**, thus confirming the transactivation results. Interestingly, the presence of the non-conjugable functional group such as the sulfate group on the side chain instead of the carboxyl end group as in 6-ECDCA (**3**) points the attention on the positive pharmacokinetic properties of compound **19** and therefore on its therapeutical potential in liver FXR mediated diseases. On the other hand, panel A in Fig. 10 shows compounds **7**, **9** and **10** completely unable to recruit SRC-1 in cell free Alpha screen assay, thus excluding any pharmacokinetic elements in their FXR inactivity on cellular luciferase assays (Fig. 7, panel A) and indirectly reaffirming compound **7** as a selective GPBAR1 agonist (Fig. 7, panel B).

RT-PCR further confirmed **19** as a dual FXR/GPBAR1 agonist and the GPBAR1 mediated pharmacological effect of compound **7**. As shown in Fig. 10 panels B–D, **19** was able to induce the expression of BSEP and OSTα, two canonical FXR targeted genes, whereas both compounds increased pro-glucagon gene expression in GLUTAg cells. The observed 2 fold of BSEP upregulation as well as the robust induction of OSTα mRNA is consistent with the activation of the FXR-mediated effect by compound **19** 39.

In summary, a series of 6-ethylcholane derivatives were designed and synthesized and all the newly synthesized compounds were evaluated *in vitro* for their activity towards FXR and GPBAR1. Concerning the structural features, α-substituents introduced at C-6 and C-7 positions play a significant role in FXR and GPBAR1 activity, with all derivatives showing this configurational disposition able to transactivate both receptors. Even if the sulfate derivative **19** is the most potent FXR agonist discovered in this study, the dual modulation is a general trend within compounds **12**–**19**, independently by the length and the functional group of the side chain as well as by the substitution at C-3. On the contrary, modification at the configurational disposition of one or both substituents on ring B is clearly deleterious in term of FXR activation but represents a promising strategy in the identification and development of selective GPBAR1 agonists with compound **7**, the most potent GPBAR1 activator identified in this study.

## Methods

### Chemistry

Specific rotations were measured on a Jasco P-2000 polarimeter. High-resolution ESI-MS spectra were performed with a Micromass Q-TOF mass spectrometer. NMR spectra were obtained on Varian Inova 400, 500 and 700 NMR spectrometers (^1^H at 400 and 700 MHz, ^13^C at 100 and 175 MHz, respectively) equipped with a SUN microsystem ultra5 hardware and recorded in CD_3_OD (δ_H_ = 3.30 and δ_C_ = 49.0 ppm) and CDCl_3_ (δ_H_ = 7.26 and δ_C_ = 77.0 ppm). All of the detected signals were in accordance with the proposed structures. Coupling constants (*J* values) are given in Hertz (Hz), and chemical shifts (δ) are reported in ppm and referred to CHD_2_OD and CHCl_3_ as internal standards. Spin multiplicities are given as s (singlet), br s (broad singlet), d (doublet), or m (multiplet). Through-space ^1^H connectivities were evidenced using NOESY experiment with mixing time of 400 ms. HPLC was performed with a Waters Model 510 pump equipped with Waters Rheodine injector and a differential refractometer, model 401. Reaction progress was monitored via thin-layer chromatography (TLC) on Alugram silica gel G/UV254 plates. Silica gel MN Kieselgel 60 (70–230 mesh) from Macherey-Nagel Company was used for column chromatography. All chemicals were obtained from Sigma-Aldrich, Inc. Solvents and reagents were used as supplied from commercial sources with the following exceptions. Tetrahydrofuran and triethylamine were distilled from calcium hydride immediately prior to use. Methanol was dried from magnesium methoxide as follow. Magnesium turnings (5 g) and iodine (0.5 g) are refluxed in a small (50–100 mL) quantity of methanol until all of the magnesium has reacted. The mixture is diluted (up to 1 L) with reagent grade methanol, refluxed for 2–3 h then distilled under nitrogen. All reactions were carried out under argon atmosphere using flame-dried glassware. The purities of compounds were determined to be greater than 95% by HPLC.

### Synthetic procedures

See the Supporting Information.

### Transactivation assay

For FXR and GP BAR1 mediated transactivations, HepG2 cells and HEK293T cells were transfected as described previously^25^. At 24 h post-transfection, cells were stimulated 18 h with 10 μM CDCA (**1**), TLCA (**2**), 6-ECDCA (**3**) and compounds **7–19**. After treatments, 20 μL of cellular lysates were read using Dual Luciferase Reporter Assay System (Promega Italia s.r.l., Milan, Italy) according manufacturer specifications using the Glomax 20/20 luminometer (Promega Italia s.r.l., Milan, Italy). To evaluate GPBAR1 mediated transactivation, HEK-293T cells were transfected with 200 ng of human pGL4.29 (Promega), a reporter vector containing a cAMP response element (CRE) that drives the transcription of the luciferase reporter gene luc2P, with 100 ng of pCMVSPORT6-human GPBAR1, and with 100 ng of pGL4.70. Dose-response curves were performed in HepG2 and HEK-293T cells transfected as described above and then treated with increasing concentrations of compounds **7** (1**–**10 μM), **13**, **14** and **19** (100 nM–25 μM). At 18 h post stimulations, cellular lysates were assayed for luciferase and Renilla activities using the Dual-Luciferase Reporter assay system (E1980, Promega). Luminescence was measured using Glomax 20/20 luminometer (Promega). Luciferase activities were normalized with Renilla activities.

### RNA isolation and RT-PCR

Total RNA was isolated from HepG2 or GLUTAg cells using the TRIzol reagent according to the manufacturer’s specifications (Invitrogen). One microgram of purified RNA was treated with DNase-I and reverse transcribed with Superscript II (Invitrogen). For Real Time PCR, 10 ng template was dissolved in 25 μL containing 200 nmol/L of each primer and 12.5 μL of 2 × SYBR FAST Universal ready mix (Invitrogen). All reactions were performed in triplicate, and the thermal cycling conditions were as follows: 2 min at 95 °C, followed by 40 cycles of 95 °C for 20 s and 60 °C for 30 s in iCycler iQ instrument (Biorad). The relative mRNA expression was calculated and expressed as 2-(ΔΔCt). Forward and reverse primer sequences were the following: human GAPDH, gaaggtgaaggtcggagt and catgggtggaatcatattggaa; human OSTα, tgttgggccctttccaatac and ggctcccatgttctgctcac; human BSEP, gggccattgtacgagatcctaa and tgcaccgtcttttcactttctg; mouse GAPDH, ctgagtatgtcgtggagtctac and gttggtggtgcaggatgcattg; mouse Pro-glucagon, tgaagacaaacgccactcac and caatgttgttccggttcctc.

### Direct interaction on FXR by Alpha screen technology in a coactivator recruitment assay

Anti-GST-coated acceptor beads were used to capture the GST-fusion FXR-LBD whereas the biotinylated-SRC-1 peptide was captured by the streptavidin donor beads. Upon illumination at 680 nm, chemical energy is transferred from donor to acceptor beads across the complex streptavidin-Donor/Src-1-Biotin/GSTFXR-LBD/Anti-GST-Acceptor and a signal is produced. The assay was performed in white, low-volume, 384-well Optiplates (PerkinElmer) using a final volume of 25 μL containing final concentrations of 10 nM of purified GST-tagged FXR-LBD protein, 30 nM biotinylated Src-1 peptide, 20 μg/mL anti-GST acceptor beads acceptor beads and 10 μg/mL of streptavidin donor bead (PerkinElmer). The assay buffer contained 50 mM Tris (pH 7.4), 50 mM KCl, 0.1% BSA, and 1 mM DTT. The stimulation times with 1 μL of tested compound (dissolved in 50% DMSO/H_2_O) were fixed to 30 min at room temperature. The concentration of DMSO in each well was maintained at a final concentration of 4%. After the addition of the detection mix (acceptor and donor beads) the plates were incubated in the dark for 4 h at room temperature and then were read in Envision microplate analyzer (PerkinElmer).

## Additional Information

**How to cite this article**: Finamore, C. *et al*. Navigation in bile acid chemical space: discovery of novel FXR and GPBAR1 ligands. *Sci. Rep.* **6**, 29320; doi: 10.1038/srep29320 (2016).

## Supplementary Material

Supplementary Information

## Figures and Tables

**Figure 1:**
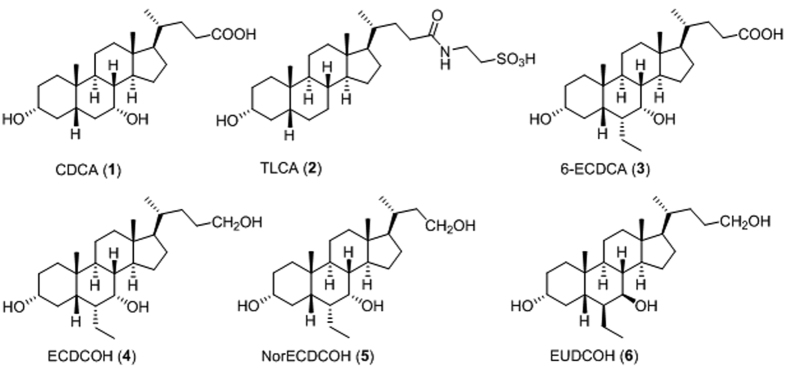
CDCA and TLCA, the most potent endogenous activators of FXR and GPBAR1, respectively. 6-ECDCA and 6α-ethylchenodeoxycholanol derivatives **4** and **5** as dual ligands and EUDCOH (**6**), a selective GPBAR1 agonist.

**Figure 2:**
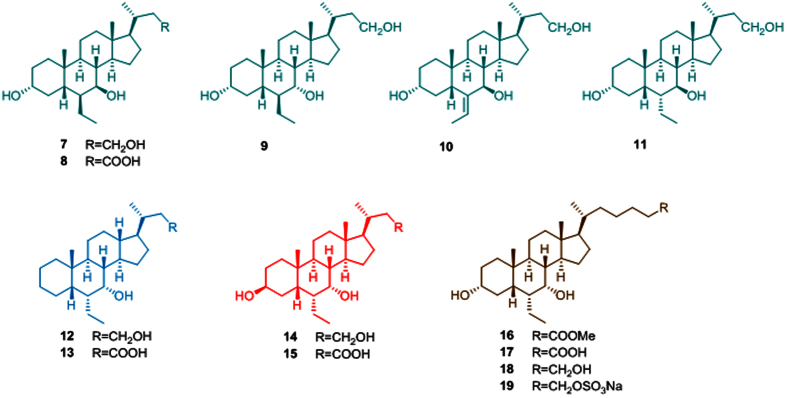
6-Ethylcholane derivatives generated in this study.
