# Supplementary material for: Navigation in bile acid chemical space: discovery of novel FXR and GPBAR1 ligands
Source: Sci Rep. 2016 Jul 6;6:29320. doi: 10.1038/srep29320 (PMC4933954; doi:10.1038/srep29320)

Supporting Information

Navigation in bile acid chemical space. Discovery of novel FXR and GPBAR1 ligands

Claudia Finamore,[1] Carmen Festa,[1] Barbara Renga,[2] Valentina Sepe,[1] Adriana Carino,[2] Dario Masullo,[1] Michele Biagioli,[2] Silvia Marchianò,[2] Angela Capolupo,[3] Maria Chiara Monti,[3] Stefano Fiorucci,[2] and Angela Zampella[1]*

[1]Department of Pharmacy, University of Naples "Federico II", Via D. Montesano, 49, 80131 Naples, Italy
[2]Department of Surgery and Biomedical Sciences, Nuova Facoltà di Medicina, P.zza L. Severi 1, 06132 Perugia, Italy
[3]Department of Pharmacy, University of Salerno, Via Giovanni Paolo II, 132, 84084 Fisciano (Salerno), Italy

*A.Z.: phone, +39 081678525; fax, +39 081678552; e-mail, angela.zampella@unina.it

Table of contents:

- Synthetic procedures S2
- ^{1}H-NMR spectrum of compound **7** S13
- ^{1}H-NMR spectrum of compound **8** S14
- ^{1}H-NMR spectrum of compound **9** S15
- ^{1}H-NMR spectrum of compound **10** S16
- ^{1}H-NMR spectrum of compound **11** S17
- ^{1}H-NMR spectrum of compound **12** S18
- ^{1}H-NMR spectrum of compound **13** S19
- ^{1}H-NMR spectrum of compound **14** S20
- ^{1}H-NMR spectrum of compound **15** S21
- ^{1}H-NMR spectrum of compound **16** S22
- ^{1}H-NMR spectrum of compound **17** S23
- ^{1}H-NMR spectrum of compound **18** S24

Synthetic procedures.

Methyl 3α-hydroxy-7-keto-24-*nor*-5β-cholan-23-oate (22). 7-KLCA (2 g, 5.12 mmol) was subjected to Beckmann degradation at C24 and methylation at C-23 furnishing **22** in 66% yield. The Beckmann degradation was previously described[1].

Methyl (*E*)-3α-acetoxy-6-ethylidene-7-keto-24-*nor*-5β-cholan-23-oate (23). At the solution of the methyl ester **22** (1.32 g, 3.38 mmol) in dry pyridine (10 mL), an excess of acetic anhydride (3.2 mL, 34mmol) was added. After 2 h, the pyridine was concentrated under vacuum. The residue was poured into cold water (100 mL) and extracted with ethyl acetate (3×50 mL). The combined organic phases were dried (Na_2SO_4) and concentrated to give a residue that was further purified by flash chromatography on silica gel using hexane/ethyl acetate 95:5 and 0.5% of triethylamine as eluent. To a solution of diisopropylamine (7.2 mL, 51.0 mmol) in dry THF (50 mL) was added dropwise a solution of *n*-butyllithium (19.0 mL, 2.5 M in hexane, 47.6 mmol) at -78 °C. After 30 min, trimethylchlorosilane (8.2 mL, 64.6 mmol) was added. After additional 30 min, a solution of residue(1.46 g, 3.4mmol) in dry THF (10 mL) was added. The reaction was stirred at -78 °C for 45 min and then triethylamine (16.6 mL, 119 mmol) was added. After 1 h, the reaction mixture was allowed to warm to -20 °C, treated with aqueous saturated solution of $NaHCO_3$ (100 mL) and brought up to room temperature in 2 h. The aqueous phase was extracted with ethyl acetate (3x50 mL). The combined organic phases were washed then with saturated solution of $NaHCO_3$, water and brine. After drying, the residue was evaporated under *vacuum* to give 2 g of residue, that was diluted in dry CH_2Cl_2 (20 mL) and cooled at -78 °C. At this stirred solution acetaldehyde (960 μL, 17.0 mmol) and $BF_3{\cdot}OEt_2$ (4.2 mL, 34mmol) were added dropwise. The reaction mixture was stirred for1 h at -60 °C and allowed to warm to room temperature. The mixture was quenched with saturated aqueous solution of $NaHCO_3$ and extracted with CH_2Cl_2. The combined organic phases were washed with brine, dried over anhydrous Na_2SO_4 and concentrated under *vacuum*. Purification by silica gel (hexane-ethyl acetate 99:1 and 0.5% TEA) gave compound **23** (940 mg, 60% over three steps).

NMR analysis demonstrated a diasteromeric ratio E/Z >95%. The *E* configuration at the exociclic double bond was established by dipolar coupling H_3-25 (δ 1.67)/H-5 (δ 2.61) in NOESY spectrum (400 MHz, mixing time 400 ms). $[\alpha]_D^{25}$= -3.31 (*c* 3.25, CH_3OH). Selected ^{1}H NMR (400 MHz, $CDCl_3$):δ 6.17 (1H, q, *J* = 7.2 Hz, H-24), 4.75 (1H, m, H-3), 3.64 (3H, s, $COOCH_3$), 2.61 (1H, dd, *J* = 13.1, 4.0 Hz, H-5), 1.98 (3H, s, $COCH_3$), 1.67 (3H, d, *J* = 7.2 Hz, H_3-25), 1.00 (3H, s, H_3-19), 0.97 (3H, d, *J* = 6.8 Hz, H_3-21), 0.67 (3H, s, H_3-18). ^{13}C NMR (100 MHz, $CDCl_3$): δ 204.5, 174.2, 170.5, 143.0, 130.6, 72.5, 54.7, 51.4, 50.7, 48.6, 45.3, 43.7, 41.5, 39.1, 38.8, 34.6, 34.2, 33.6, 33.4, 28.5, 25.9 (2C), 22.8, 21.3 (2C), 19.7, 12.7, 12.1. HR ESIMS *m/z* 459.3107 $[M+H]^+$, $C_{28}H_{43}O_5$ requires 459.3110.

Methyl 3α-acetoxy-6β-ethyl-7-keto-24-*nor*-5β-cholan-23-oate (24). A solution of **23** (600 mg, 1.3 mmol) in THF dry/MeOH dry (100 mL, 1:1 v/v) was hydrogenated in presence of $Pd(OH)_2$ 20% wt on activated carbon (100 mg) degussa type. The mixture was transferred to a standard PARR apparatus and flushed with nitrogen and hydrogen several times. The apparatus was shacked under 50 psi of H_2. The reaction was stirred at room temperature for 12 h. The catalyst was filtered through Celite, and the recovered filtrate was concentrated under vacuum to give **24** (600 mg, quantitative yield). The β configuration of ethyl group at C-6 was determined by dipolar couplings H_3-25 (δ 0.83)/ H_3-19 (δ 1.22) in NOESY spectrum (400 MHz, mixing time 400 ms). $[\alpha]_D^{25}$=+10.4 (*c* 0.39, CH_3OH). Selected ^{1}H NMR (400 MHz, $CDCl_3$): δ 4.65 (1H, m, H-3), 3.67 (3H, s, $COOCH_3$), 2.60 (1H, t, *J* = 11.2 Hz, H-8), 2.43 (1H, dd, *J* = 14.2, 2.6 Hz, H-22a), 1.98 (3H, s, $COCH_3$), 1.88 (1H, m ovl, H-6), 1.22 (3H, s, H_3-19), 0.98 (3H, d, *J* = 6.4 Hz, H_3-21), 0.83 (3H, t, *J* = 7.0 Hz, H_3-25), 0.70 (3H, s, H_3-18). ^{13}C NMR (100 MHz, $CDCl_3$): δ 215.3, 174.0, 170.5, 72.8, 61.9, 55.0, 51.4, 49.2, 48.7, 45.5, 42.9, 42.6, 41.4, 38.7 (2C), 35.6, 35.3, 34.9, 28.3 (2C), 26.5, 25.9, 24.8, 21.4, 21.3, 19.6, 13.0, 12.1. HR ESIMS *m/z* 461.3265 $[M+H]^+$, $C_{28}H_{45}O_5$ requires 461.3267.

6β-ethyl-3α,7β-hydroxy-24-*nor*-5β-cholan-23-ol (7) and 6β-ethyl-3α,7α-hydroxy-24-*nor*-5β-cholan-23-ol (9). A methanol solution of compound **24** (100 mg, 0.22 mmol), a large excess of

$NaBH_4$ was added at 0 °C. The mixture was left at room temperature for 10 h and then water and MeOH was added dropwise during a period of 15 min at 0 °C with effervescence being observed. Then after evaporation of the solvents, the residue was diluted with water and extracted with ethyl acetate (3 x 50 mL). The combined extract was washed with brine, dried with Na_2SO_4, and evaporated to give 90 mg of a crude residue that was subjected to the next step without further purification. To a solution of crude residue (90 mg, 0.214 mmol) in dry THF (15 mL), at 0 °C dry methanol (60 µL, 1.49 mmol) and $LiBH_4$ (749 µL, 2 M in THF, 1.49 mmol) were added. The resulting mixture was stirred for 2 h at 0 °C. The mixture was quenched by addition of 1 M NaOH (428 µL) and then ethyl acetate. The organic phase was washed with water, dried (Na_2SO_4) and concentrated. HPLC purification on a Nucleodur 100-5 C18 (5 µm; 10 mm i.d. x 250 mm) with $MeOH/H_2O$ (86:14) as eluent (flow rate 3 mL/min), gave 47 mg of **7** (54%, t_R= 11 min) and 20 mg of **9** (23%, t_R= 15 min).

6β-ethyl-3α,7β-hydroxy-24-*nor*-5β-cholan-23-ol (7). $[\alpha]_D^{25}$=+11.57 (*c* 0.14, CH_3OH) .Selected ^1H NMR (400 MHz, CD_3OD): δ 3.73 (1H, dd, *J* = 10.5, 5.5 Hz, H-7), 3.61 (1H, m, H-23a), 3.51 (1H, m, ovl, H-23b), 3.51 (1H, m, ovl, H-3), 0.98 (3H, d, ovl, H_3-21), 0.97 (3H, s, H_3-19), 0.96 (3H, t, ovl, H_3-25), 0.70 (3H, s, H_3-18). ^{13}C NMR (100 MHz, CD_3OD): δ 75.2, 71.8, 60.8, 57.5, 56.6, 51.5, 45.5, 44.8, 42.0, 41.4, 40.7, 40.3, 39.9, 36.9, 36.0, 34.2, 30.5, 29.6, 28.3, 26.2, 23.4, 22.0, 19.4, 14.7, 12.9. HR ESIMS *m/z* 393.3365 $[M+H]^+$, $C_{25}H_{45}O_3$ requires 393.3369.

6β-ethyl-3α,7α-hydroxy-24-*nor*-5β-cholan-23-ol (9). $[\alpha]_D^{25}$= + 9.16 (*c* 0.62, CH_3OH). Selected ^1H NMR (400 MHz, CD_3OD): δ 3.63 (1H, m, H-23a), 3.60 (1H, m, H-7), 3.55 (1H, m, H-23b), 3.37 (1H, m, H-3), 2.30 (1H, q, *J* = 12.5 Hz, H-4a), 0.97 (3H, d, *J* = 6.6 Hz, H_3-21), 0.95 (3H, s, H_3-19), 0.95 (3H, t, *J* = 7.0 Hz, H_3-25), 0.72 (3H, s, H_3-18). ^{13}C NMR (100 MHz, CD_3OD): δ 72.8, 72.7, 60.8, 57.9, 52.7, 51.4, 47.5, 43.7, 42.3, 41.0, 39.9, 37.5, 37.3, 36.7, 34.2, 33.3, 31.0, 29.6, 29.4, 26.2, 24.8, 21.6, 19.3, 14.5, 12.1. HR ESIMS *m/z* 393.3367 $[M+H]^+$, $C_{25}H_{45}O_3$ requires 393.3369.

6β-ethyl-3α,7β-hydroxy-24-*nor*-5β-cholan-23-oic acid (8). To a methanol solution of compound **24** (200 mg, 0.44 mmol), a large excess of $NaBH_4$ was added at 0 °C. The mixture was left at room temperature for 2 h and then water and MeOH were added dropwise during a period of 15 min at 0 °C with effervescence being observed. After evaporation of the solvents, the residue was diluted with water and extracted with ethyl acetate (3 x 50 mL).The combined extract was washed with brine, dried with Na_2SO_4 and evaporated to give 216 mg of **25** that was subjected to the next step without further purification. Compound **25** (216 mg, 0.52 mmol) was hydrolyzed with NaOH (207 mg, 5.17 mmol) in a solution of MeOH:H_2O 1:1 v/v (20 mL). The resulting solution was then concentrated under vacuum, diluted with water, acidified with HCl 6 N and extracted with ethyl acetate (3 x 50 mL). The collected organic phases were washed with brine, dried over Na_2SO_4 anhydrous and evaporated under reduced pressure. HPLC purification on a Nucleodur 100-5 C18 (5 μm; 10 mm i.d. x 250 mm) with MeOH/H_2O (86:14) as eluent (flow rate 3 mL/min), gave compound **8** (147 mg, 0.36 mmol) as a white solid (82%, t_R=9 min). $[\alpha]_D^{25}$= +23.7 (*c* 0.14, CH_3OH). Selected ^1H NMR (400 MHz, CD_3OD): δ 3.74 (1H, dd, *J* = 10.1, 5.7 Hz, H-7), 3.50 (1H, m, ovl, H-3), 2.42 (1H, d, *J* = 11.0 Hz, H-22a), 1.02 (3H, d, ovl, H_3-21), 1.00 (3H, s, H_3-19), 0.96 (3H, t, *J*= 7.0 Hz, H_3-25), 0.76 (3H, s, H_3-18). HR ESIMS *m/z* 405.3001 $[M-H]^-$, $C_{25}H_{41}O_4$ requires 405.3005.

6-ethylidene-3α,7β-dihydroxy-24-*nor*-5β-cholan-23-ol (10). Compound **23** (100 mg, 0.22 mmol) was subjected to the same operative condition previously described for compounds **7** and **9**. HPLC purification on a Nucleodur 100-5 C18 (5 μm; 10 mm i.d. x 250 mm) with MeOH/H_2O (86:14) as eluent (flow rate 3 mL/min), gave 73 mg of compound **10** (t_R= 8 min, 85% over two steps). $[\alpha]_D^{25}$= +20.6 (*c* 2.43, CH_3OH). Selected^1H NMR (400 MHz, CD_3OD): δ 5.66 (1H, q, *J* = 6.8 Hz, H-24), 3.92 (1H, d, *J* = 9.8 Hz, H-7), 3.60 (1H, m, H-23a), 3.56 (1H, m, H-3), 3.55 (1H, m, H-23b), 2.52 (1H, dd, *J* = 3.7, 13.2 Hz, H-5), 1.63 (3H, d, *J* = 6.8 Hz, H_3-25), 0.98 (3H, d, *J* = 6.5 Hz, H_3-21), 0.95 (3H, s, H_3-19), 0.71 (3H, s, H_3-18). ^{13}C NMR (100 MHz, CD_3OD):δ 143.7, 115.4, 74.1, 71.8,

60.8, 58.0, 57.1, 46.1, 45.9, 45.1, 41.6, 41.1, 39.9, 37.0, 36.4, 35.8, 34.1, 30.9, 29.8, 28.1, 23.5, 22.5, 19.5, 12.7, 12.6. HR ESIMS *m/z* 391.3208 $[M+H]^+$, $C_{25}H_{43}O_3$ requires 391.3212.

6α-ethyl-3α,7β-dihydroxy-24-*nor*-5β-cholan-23-ol (11). A solution of **10** (30 mg, 0.076 mmol) in THF dry/MeOH dry (5 mL, 1:1 v/v) was hydrogenated in presence of $Pd(OH)_2$ 20% wt on activated carbon (10 mg) degussa type. The reaction was stirred at room temperature for 12 h. The catalyst was filtered through Celite, and the recovered filtrate was concentrated under vacuum to give **11** (30 mg, quantitative yield). $[\alpha]_D^{25}$= -3.3 (*c* 0.10, CH_3OH). Selected ^{1}H NMR (400 MHz, CD_3OD): δ 3.62 (1H, m, H-23a), 3.54 (1H, m, H-23b), 3.45 (1H, m, H-3), 3.08 (1H, t, *J* = 9.8 Hz, H-7), 0.97 (3H, d, *J* = 6.5 Hz, H_3-21), 0.95 (3H, s, H_3-19), 0.86 (3H, t, *J* = 7.4 Hz, H_3-25), 0.73 (3H, s, H_3-18). ^{13}C NMR (100 MHz, CD_3OD): δ 76.4, 72.5, 60.8, 57.9, 57.2, 46.2, 45.1, 44.7, 41.8, 41.2, 40.0, 39.8, 36.5, 35.6, 34.2, 31.2, 30.9, 29.9, 27.9, 24.1, 22.7, 22.0, 19.5, 12.7, 11.7. HR ESIMS *m/z* 393.3365 $[M+H]^+$, $C_{25}H_{45}O_3$ requires 393.3369.

Methyl 3α-toxyloxy-6α-ethyl-7-keto-24-nor-5β-cholan-23-oate (26). Compound **24** (300 mg, 0.65 mmol) was treated with 0.5 M MeONa (13 mL) in dry methanol and stirred at room temperature over night, in order to obtain the deacetylation at C-3 and inversion at C-6. Then after addition of water and evaporation of the solvents, the residue was diluted with water and extracted with ethyl acetate (3 x 50 mL). The combined extract was washed with brine, dried with Na_2SO_4 and evaporated to give 240 mg of a crude residue that was subjected to the next step without further purification. To a solution of a crude residue (240 mg, 0.52 mmol) in dry pyridine (10 mL), tosyl chloride (1.11 g, 12.2 mmol) was added, and the mixture was stirred at room temperature for 6 h. It was poured into cold water (30 mL) and extracted with CH_2Cl_2 (3 × 30 mL), to give 270 mg of **26** (73% over two steps) that was subjected to the next step without further purification.

6α-ethyl-7α-hydroxy-24-*nor*-5β-cholan-23-ol (12). Lithium bromide (50 mg, 0.58 mmol) and lithium carbonate (42.2 mg, 0.58 mmol) were added to a solution of compound **26** (150 mg, 0.26 mmol) in dry DMF (10 mL), and the mixture was refluxed for 2 h. After cooling to room

temperature, the mixture was slowly poured into 10% HCl solution (10 mL) and extracted with CH_2Cl_2 (3 × 30 mL). The combined organic layer was washed successively with water, saturated $NaHCO_3$ solution and water, and then dried over anhydrous $MgSO_4$ and evaporated to dryness to give 145 mg of oily residue (quantitative yield), that was subjected to next step without any purification. An oven-dried 10 mL flask was charged with 10% palladium on carbon (5 mg) and the product obtained (145 mg, 0.25 mmol) and the flask was evacuated and flushed with argon. Absolute methanol (5 mL) and dry THF (5 mL) were added, and the flask was flushed with hydrogen. The reaction was stirred at room temperature under H_2 (1 atm) over night. The mixture was filtered through celite, and the recovered filtrate was concentrated to give 110 mg of **27** (0.026 mmol, quantitative yield over two steps). Dry methanol (35 μL, 0.87 mmol) and $LiBH_4$ (435 μL, 2 M in THF,0.87 mmol) were added to a solution of **27** (50 mg, 0.124 mmol) in dry THF (5 mL) at 0 °C under argon and the resulting mixture was stirred for 3 h at 0 °C. The mixture was quenched by addition of NaOH (1 M, 248μL) and then allowed to warm to room temperature. Ethyl acetate was added and the separated aqueous phase was extracted with ethyl acetate (3 × 15 mL). The combined organic phases were washed with water, dried (Na_2SO_4) and concentrated. HPLC purification on a Nucleodur 100-5 C18 (5 μm; 5 mm i.d. x 250 mm) with MeOH/H_2O (9:1) as eluent (flow rate 1 mL/min), gave 32.5 mg of **12** (t_R= 17.2 min, 70%). $[\alpha]_D^{25}$= +1.0 (*c* 2.15, CH_3OH). Selected^{1}H NMR (400 MHz, CD_3OD): δ 3.66 (1H, br s, H-7), 3.61 (1H, m, H-23a), 3.54 (1H, m, H-23b), 0.96 (3H, d, *J* = 6.7 Hz, H_3-21), 0.91 (3H, s, H_3-19), 0.89 (3H, t, *J* = 7.3 Hz, H_3-25), 0.70 (3H, s, H_3-18). ^{13}C NMR (100 MHz, CD_3OD):δ 71.5, 60.8, 57.8, 51.7, 48.6, 43.8, 43.3, 41.5, 41.1, 39.9, 39.2, 37.4, 34.5, 34.2, 29.4, 28.8, 25.0, 24.6 (2C), 23.5, 22.5, 22.0, 19.4, 12.2, 12.1. HR ESIMS *m/z* 377.3423 $[M+H]^+$, $C_{25}H_{45}O_2$ requires 377.3420.

6α-ethyl-7α-hydroxy-24-*nor*-5β-cholan-23-oic acid (13). Compound **27** (60 mg, 0.15 mmol) was hydrolyzed with NaOH (57.6 mg, 1.44 mmol) in a solution of MeOH:H_2O 1:1 v/v (6 mL) on reflux for 4 h. Crude carboxylic acid intermediate (65 mg, 0.17 mmol) was treated with $LiBH_4$ by

analogous procedures to those detailed above for compound **12**. Purification by silica gel (CH_2Cl_2-MeOH 99:1) furnished 53.5 mg of compound **13** (0.14 mmol, 92%). $[\alpha]_D^{25}$= +1.5 (*c* 0.27, CH_3OH). Selected ^1H NMR (400 MHz, CD_3OD): δ 3.64 (1H, br s, H-7), 2.41 (1H, dd, *J* = 11.0, 2.6 Hz, H-22a), 1.00 (3H, d, *J* = 6.0 Hz, H_3-21), 0.90 (3H, s, H_3-19), 0.87 (3H, t, *J* = 7.4 Hz, H_3-25), 0.71 (3H, s, H_3-18). ^{13}C NMR (100 MHz, CD_3OD): δ 178.9, 71.6, 57.5, 51.7, 48.6, 43.8, 43.4, 43.3, 41.5, 40.9, 39.1, 37.4, 35.2, 34.6, 29.4, 28.8, 25.0, 24.6 (2C), 23.5, 22.4, 22.0, 20.1, 12.2, 12.1. HR ESIMS *m/z* 389.3052 $[M-H]^-$, $C_{25}H_{41}O_3$ requires 389.3056.

Methyl 3β-hydroxy-6α-ethyl-7-keto-24-nor-5β-cholan-23-oate (28). A solution of intermediate **26** (100 mg, 0.17 mmol) and CH_3COOK (18 mg, 0.18 mmol) dissolved in water (1 mL) and N,N'-dimethylformamide (DMF, 4 mL), was refluxed for 3 h. The solution was cooled at room temperature and then ethyl acetate and water were added. The separated aqueous phase was extracted with ethyl acetate (3 × 30 mL). The combined organic phases were washed with water, dried (Na_2SO_4) and evaporated to dryness to give intermediate **28** (100 mg) that was used as starting material in the synthesis of compounds **14** and **15** without further purification.

6α-ethyl-3β,7α-dihydroxy-24-*nor*-5β-cholan-23-ol (14). Compound **28** (60 mg,0.14 mmol) was subjected at $LiBH_4$ reduction with the same procedures previous described for compound **12**, to give 55 mg of a crude residue. HPLC purification on a Nucleodur 100-5 C18 (5 μm; 5 mm i.d. x 250 mm) with MeOH/H_2O (8:2) as eluent (flow rate 1 mL/min), gave 31 mg of **14** (t_R= 14min, 57%). $[\alpha]_D^{25}$= +7.1 (*c* 1.23, CH_3OH). Selected ^1H NMR (400 MHz, CD_3OD): δ 3.97 (1H, br s, H-3), 3.67 (1H, br s, H-7), 3.61 (1H, m, H-23a), 3.55 (1H, m, H-23b), 0.97 (3H, d, *J* = 6.6 Hz, H_3-21), 0.95 (3H, s, H_3-19), 0.91 (3H, t, *J* = 7.4 Hz, H_3-25), 0.71 (3H, s, H_3-18). ^{13}C NMR (100 MHz, CD_3OD): δ 71.4, 67.4, 60.8, 57.9, 51.8, 43.8, 42.8, 41.5, 41.2, 41.1, 39.9, 37.0, 34.2, 33.8, 31.3, 31.2, 29.4, 28.3, 24.6, 24.2, 23.3, 22.3, 19.4, 12.2, 12.1. HR ESIMS *m/z* 393.3365 $[M+H]^+$, $C_{25}H_{45}O_3$ requires 393.3369.

6α-ethyl-3β,7α-dihydroxy-24-*nor*-5β-cholan-23-oic acid (15). Compound **28** (40 mg, 0.095 mmol) was hydrolyzed with sodium hydroxide in a solution of MeOH: H_2O 1:1 (5 mL) overnight under

reflux. The resulting solution was then concentrated under vacuum, diluted with water, acidified with HCl 6 N and extracted with ethyl acetate (3 x 50 mL). The collected organic phases were washed with brine, dried over Na_2SO_4 anhydrous and evaporated under reduced pressure, to give a crude residue (35 mg, 0086 mmol) that was subjected at $LiBH_4$ reduction, with the same procedures previous described for compound **12**. HPLC purification on a Nucleodur 100-5 C18 (5 μm; 5 mm i.d. x 250 mm) with MeOH/H_2O (88:12) as eluent (flow rate 3 mL/min), gave 22.5 mg of **15** as a white solid (58% over two steps, t_R= 9 min). $[\alpha]_D^{25}$= +2.0 (*c* 0.47, CH_3OH). Selected ^{1}H NMR (400 MHz, CD_3OD): δ 3.97 (1H, br s, H-3), 3.66 (1H, br s, H-7), 2.42 (1H, dd, *J* = 11.3, 3.3 Hz, H-22a), 1.02 (3H, d, *J* = 6.0 Hz, H_3-21), 0.94 (3H, s, H_3-19), 0.91 (3H, t, *J* = 7.3 Hz, H_3-25), 0.73 (3H, s, H_3-18). ^{13}C NMR (100 MHz, CD_3OD): δ 177.7, 71.3, 67.4, 57.4, 51.8, 43.8 (2C), 42.8, 41.5, 41.2, 41.0, 37.0, 35.1, 33.8, 31.3, 31.2, 29.4, 28.3, 24.6, 24.2, 23.3, 22.2, 20.0, 12.2, 12.1. HR ESIMS *m/z* 405.3003 $[M-H]^-$, $C_{25}H_{41}O_4$ requires 405.3005.

6α-ethyl-3α,7α-di(*tert*-butyldimethylsilyloxy)-5β-cholan-24-ol (30). 2,6-lutidine (5.35 mL, 46 mmol) and *tert*-butyldimethylsilyltrifluoromethanesulfonate (3.15 mL, 13.7 mmol) were added at 0 °C to a solution of compound **29** , prepared as previously reported[1], (1 g, 2.3 mmol) in 30 mL of CH_2Cl_2. After 2 h stirring at 0 °C, the reaction was quenched by addition of aqueous $NaHSO_4$ (1M, 100 mL). The layers were separated and the aqueous phase was extracted with CH_2Cl_2 (3×50 mL). The combined organic layers were washed with $NaHSO_4$, water, saturated aqueous $NaHCO_3$, and brine and evaporated in vacuo to give 2.2 g of methyl 6α-ethyl-3α, 7α–di(*tert*-butyldimethylsilyloxy)-5β-cholan-24-oate in the form of colourless needles, that was subjected to next step without any purification. To a solution of methyl ester (2.2 g, 3.4 mmol) in dry THF (30 mL), at 0 °C dry methanol (412.6 μL, 10.2 mmol) and $LiBH_4$ (5.1 mL, 2M in THF, 10.2 mmol) was added. The resulting mixture was stirred for 2 h at 0 °C. The mixture was quenched by addition of 1M NaOH (6.8 mL) and then ethyl acetate. The organic phase was washed with water, dried

(Na_2SO_4) and concentrated. Purification by silica gel (hexane/ethyl acetate 99:1 and 0.5% TEA) gave **30** as a white solid (1 g, 68% over two steps).

Methyl 6α-ethyl-3α,7α-di(tert-butyldimethylsilyloxy)-25,26-bis-*homo*-5β-chol-24-en-26-oate (31). DMSO (1.56 mL, 22 mmol) was added dropwise for 15 min to a solution of oxalyl chloride (5.49 mL, 11 mmol) in dry dichloromethane (30 mL) at -78 °C under argon atmosphere. After 30 min a solution of alchol **30** (1 g, 1.57 mmol) in dry CH_2Cl_2 was added via cannula and the mixture was stirred at -78 °C for 30 min. Et_3N (2.18 mL, 15.7 mmol) was added dropwise. After 1 h methyl(triphenylphosphoranylidene)acetate (1.64 g, 4.7 mmol) was added and the mixture was allowed to warm to room temperature. NaCl saturated solution was added and the aqueous phase was extracted with diethyl ether (3×50 mL). The combined organic phases were washed with water, dried (Na_2SO_4) and concentrated. Purification by silica gel (hexane-ethyl acetate 995:5 and 0.5% TEA) gave compound **31** as a colourless oil (850 mg, 79%).

Methyl 6α-ethyl-3α,7α-dihydroxy-25, 26-bis-*homo*-5β-cholan-26-oate (16). A solution of compound **31** (850 mg, 1.23 mmol) in THF dry/MeOH dry (5 mL/5 mL, v/v) was hydrogenated in presence of $Pd(OH)_2$ 5% wt on activated carbon Degussa type (5 mg). The flask was evacuated and flushed first with argon and then with hydrogen. After 12 h, the reaction was complete. The catalyst was filtered through celite, and the recovered filtrate was concentrated under vacuum to give the methyl ester, which was dissolved in methanol (40 mL). At the solution was added 1 mL of HCl 37% v/v. After 1h, silver carbonate was added at the solution to precipitate chloride. Then the reaction mixture was centrifuged and the supernatant was concentrated *in vacuo* to give compound **16** as colourless amorphous solids (500 mg, 88%). An analytic sample was purified by HPLC on a Nucleodur 100-5 C18 (5 μm; 4.6 mm i.d. x 250 mm) with MeOH/H_2O (98:2) as eluent (flow rate 1 mL/min), to give compound **16** (t_R=7min). $[\alpha]_D^{25}$= +6.85 (*c* 0.14, CH_3OH). Selected ^1H NMR (400 MHz, CD_3OD): δ 3.66 (3H, s, COOCH3), 3.65 (1H, ovl, H-7), 3.31 (1H, ovl, H-3), 2.32 (2H, t, *J* = 7.71 Hz, H_2-25), 0.94 (3H, d, *J* = 6.4 Hz, H_3-21), 0.92 (3H, s, H_3-19), 0.91 (3H, t, *J* = 6.8 Hz, H_3-28),

0.69 (3H, s, H_3-18). ^{13}C NMR (175 MHz, CD_3OD): δ 176.0, 73.2, 71.2, 57.7, 51.9, 51.7, 47.0, 43.7, 43.1, 41.6, 41.1, 37.1, 36.9, 36.8, 36.6, 34.5, 34.4 (2C), 31.3, 29.4, 26.7, 26.4, 24.6, 23.8, 23.5, 22.0, 19.2, 12.3, 12.1.HR ESIMS *m/z* 463.3783 $[M+H]^+$, $C_{29}H_{51}O_4$ requires 463.3787

6α-ethyl-3α, 7α-dihydroxy-25, 26-bis-*homo*-5β-cholan-26-oic acid (17). Compound **16** (200 mg, 0.43 mmol) was hydrolyzed with sodium hydroxide in a solution of MeOH: H_2O 1:1 (10 mL) overnight under reflux. The resulting solution was then concentrated under vacuum, diluted with water, acidified with HCl 6 N and extracted with ethyl acetate (3 x 50 mL). The collected organic phases were washed with brine, dried over Na_2SO_4 anhydrous and evaporated under reduced pressure. HPLC purification on a Nucleodur 100-5 C18 (5 μm; 4.6 mm i.d. x 250 mm) with MeOH/H_2O (92:8) as eluent (flow rate 1 mL/min), gave 172 mg of **17** as a white solid (89%, t_R= 7 min). $[\alpha]_D^{25}$= - 0.72 (*c* 0.08, CH_3OH). Selected ^1H NMR (400 MHz, CD_3OD): δ 3.66 (1H, br s, H-7), 3.31 (1H, m ovl, H-3), 2.24 (2H, t, *J* = 7.3 Hz, H_2-25), 0.95 (3H, d, *J* = 6.4 Hz, H_3-21), 0.92 (3H, s, H_3-19), 0.91 (3H, t, *J* = 6.9 Hz, H_3-28), 0.70 (3H, s, H_3-18). ^{13}C NMR (175 MHz, CD_3OD): δ 186.7, 73.3, 71.3, 57.7, 51.7, 47.0, 43.7, 43.1, 41.6, 41.1, 37.1, 36.9, 36.8, 36.6, 34.5, 34.4 (2C), 31.3, 29.4, 27.0 (2C), 24.6, 23.8, 23.5, 22.0, 19.2, 12.2, 12.0. HR ESIMS *m/z* 447.3469 $[M-H]^-$, $C_{28}H_{47}O_4$ requires 447.3474.

6α-ethyl-3α, 7α-dihydroxy-25, 26-bis-*homo*-5β-cholan-26-ol (18). Compound **16** (300 mg, 0.65 mmol) was subjected to a $LiBH_4$ reduction with the same procedure previous described for compound **30**, to give 280 mg of crude residue. HPLC purification on a Nucleodur 100-5 C18 (5 μm; 4.6 mm i.d. x 250 mm) with MeOH/H_2O (9:1) as eluent (flow rate 1 mL/min), gave 220 mg of **18** (78%, t_R= 14 min). $[\alpha]_D^{25}$= +10.6 (*c* 0.25, CH_3OH). Selected ^1H NMR (400 MHz, CD_3OD): δ 3.64 (1H, br s, H-7), 3.53 (2H, t, *J* = 6.6 Hz, H_2-26), 3.30 (1H, m ovl, H-3), 0.94 (3H, d, *J* = 6.7 Hz, H_3-21), 0.91 (3H, s, H_3-19), 0.90 (3H, t, *J* = 7.0 Hz, H_3-28), 0.68 (3H, s, H_3-18). ^{13}C NMR (175 MHz, CD_3OD): δ 73.3, 71.3, 63.1, 57.7, 51.7, 47.0, 43.7, 43.1, 41.6, 41.1, 37.1, 36.8 (2C), 36.6,

34.5, 34.4, 33.7, 31.3, 29.5, 27.4, 27.0, 24.6, 23.8, 23.5, 22.0, 19.3, 12.3, 12.0. HR ESIMS *m/z* 435.3835 $[M+H]^+$, $C_{28}H_{51}O_3$ requires 435.3838.

6α-ethyl-3α,7α-dihydroxy-25,26-bis-*homo*-5β-cholan-26-yl-26-sodium sulfate (19). The triethylamine-sulfur trioxide complex (65.3 mg, 0.36 mmol) was added to a solution of compound **18** (50 mg, 0.12 mmol) in DMF dry (3 mL) under an argon atmosphere, and the mixture was stirred at 70°C for 24 h. The solvent was concentrated *in vacuo*. The residue was poured over a RP18 column. Fraction eluted with MeOH 100% gave a mixture that was further purified by HPLC on a Nucleodur 100-5 C18 (5 μm; 4.6 mm i.d. x 250 mm) with MeOH/H_2O (78:22) as eluent (flow rate 1 mL/min), to give 16 mg (0.030 mmol, 25%) of compound **19** (t_R=16 min). $[\alpha]_D^{25}$= -6.14 (*c* 0.07, CH_3OH). ^1H NMR (400 MHz, CD_3OD): δ 3.99 (2H, t, *J* = 6.6 Hz, H_2-26), 3.65 (1H, br s, H-7), 3.31 (1H, m ovl, H-3), 0.94 (3H, d, *J* = 6.2 Hz, H_3-21), 0.91 (3H, s, H_3-19), 0.90 (3H, t, *J* = 7.0 Hz, H_3-28), 0.69 (3H, s, H_3-18). HR ESIMS *m/z* 513.3247 $[M-Na]^-$, $C_{28}H_{49}O_6S$ requires 513.3250.

[1] D'Amore, C. *et al*. Design, synthesis, and biological evaluation of potent dual agonists of nuclear and membrane bile acid receptors. *J. Med. Chem.***57**, 937-954 (2014).

^{1}H NMR (400 MHz, CD_3OD) of compound **7**

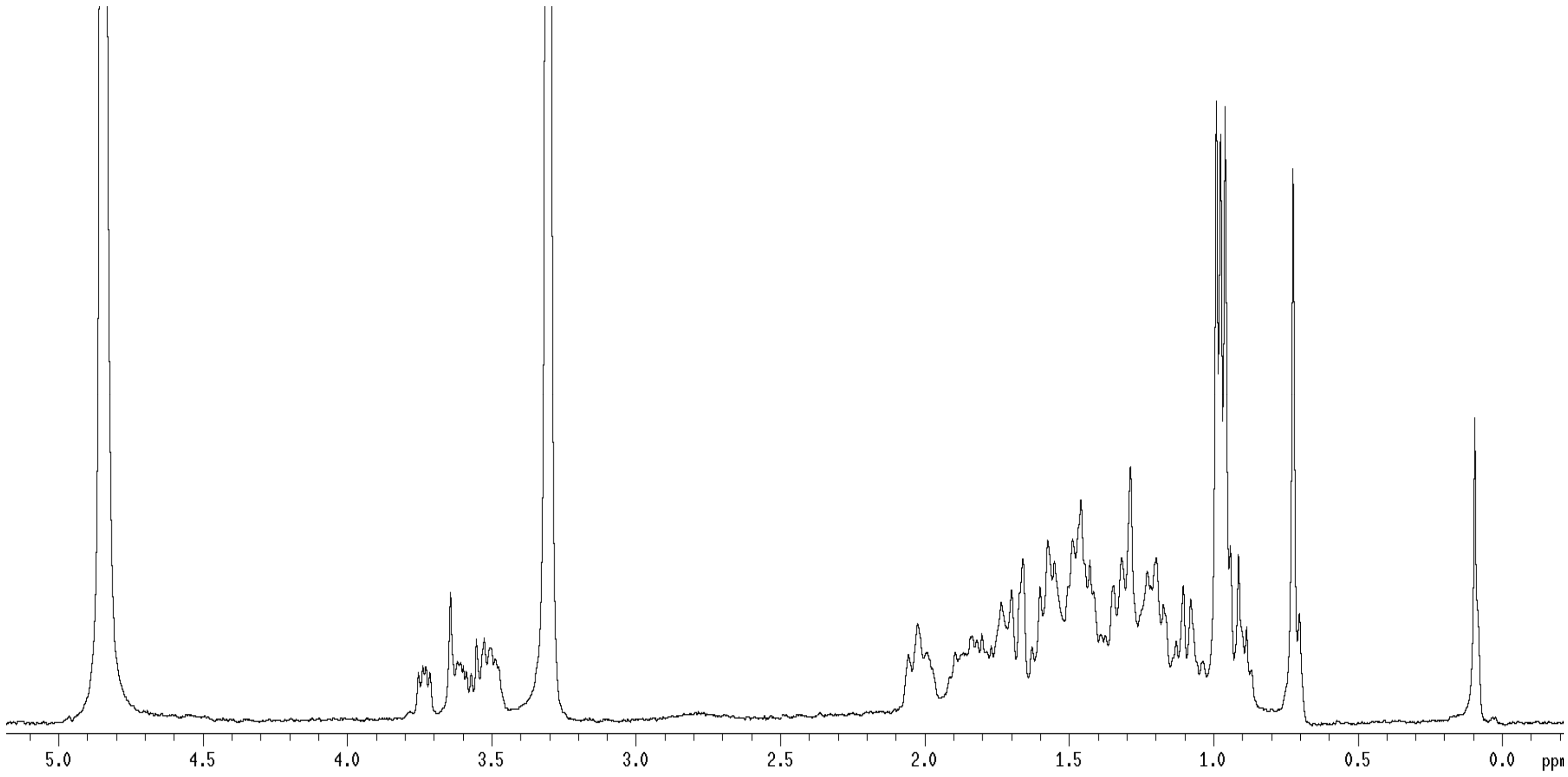

^{1}H NMR (400 MHz, CD_3OD) of compound **8**

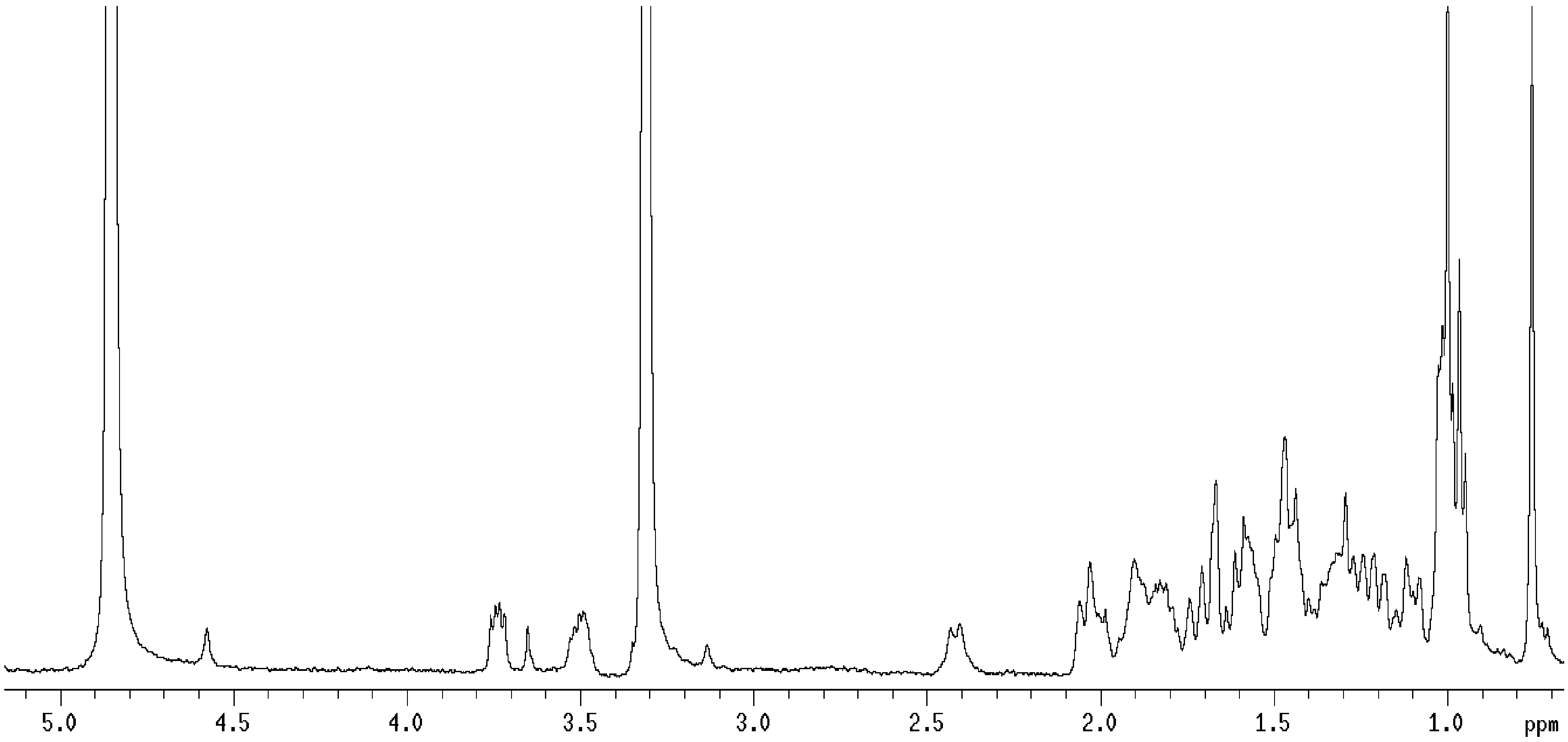

^{1}H NMR (400 MHz, CD_3OD) of compound **9**

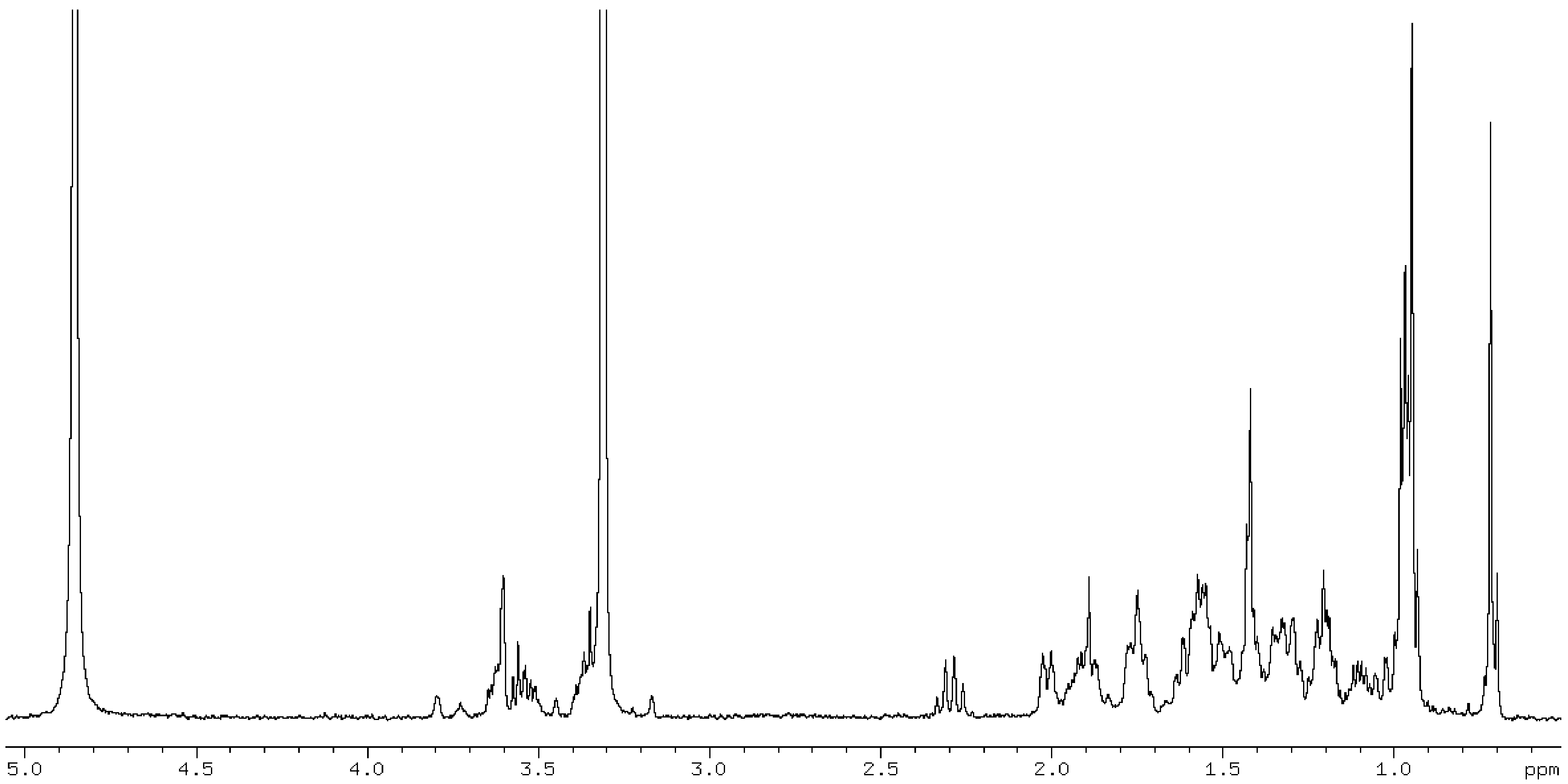

^{1}H NMR (400 MHz, CD_3OD) of compound **10**

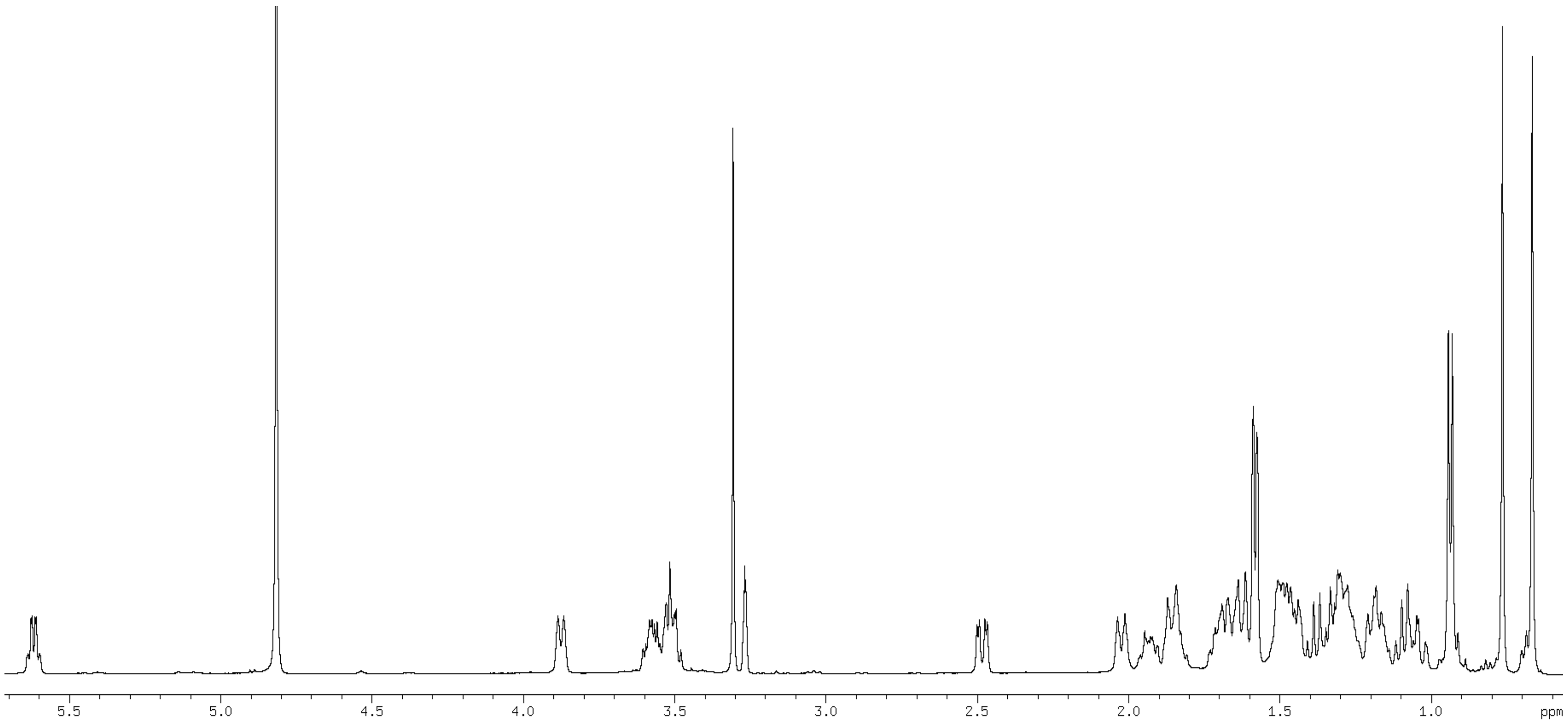

^{1}H NMR (400 MHz, CD_3OD) of compound **11**

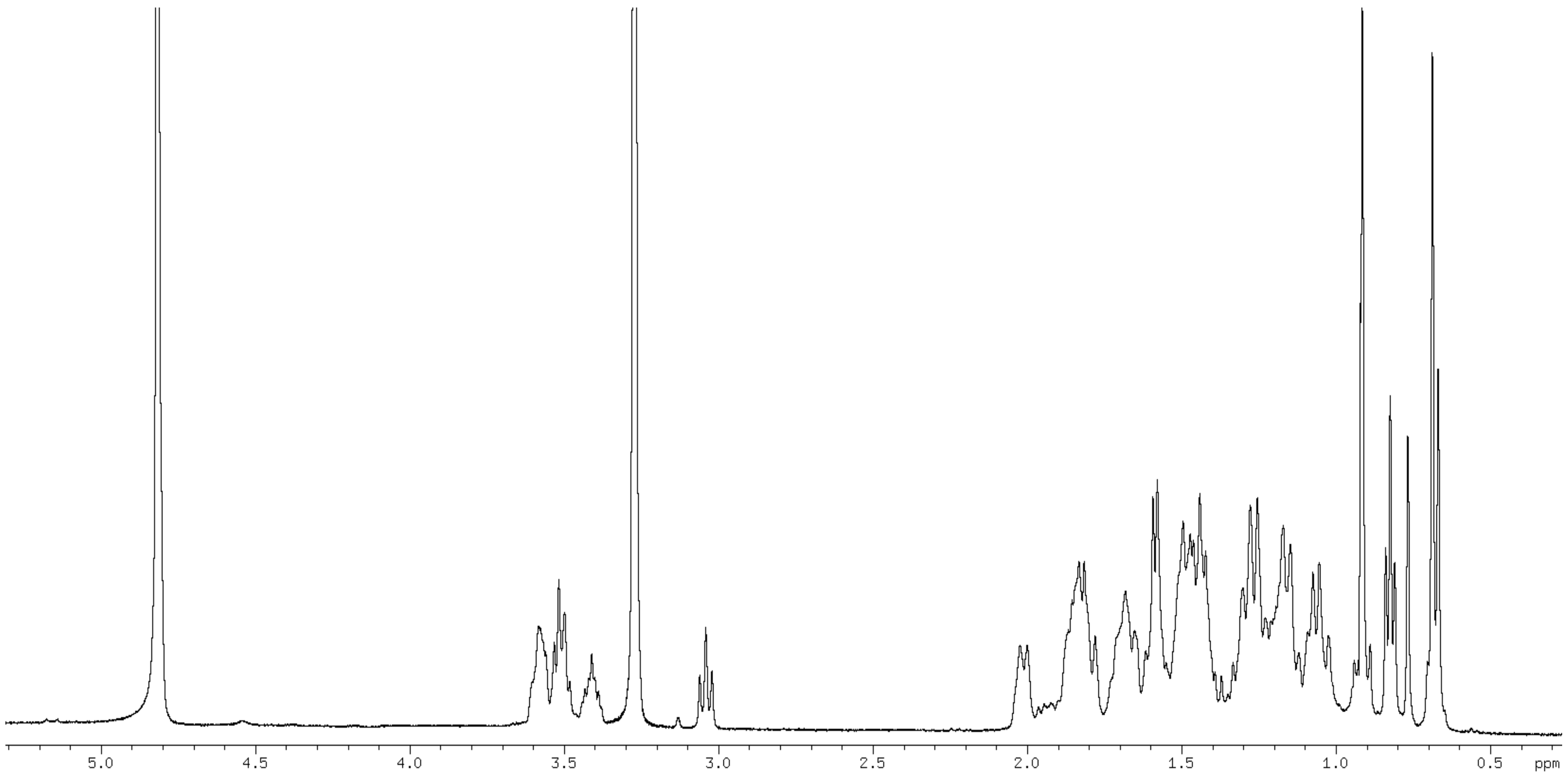

^{1}H NMR (400 MHz, CD_3OD) of compound **12**

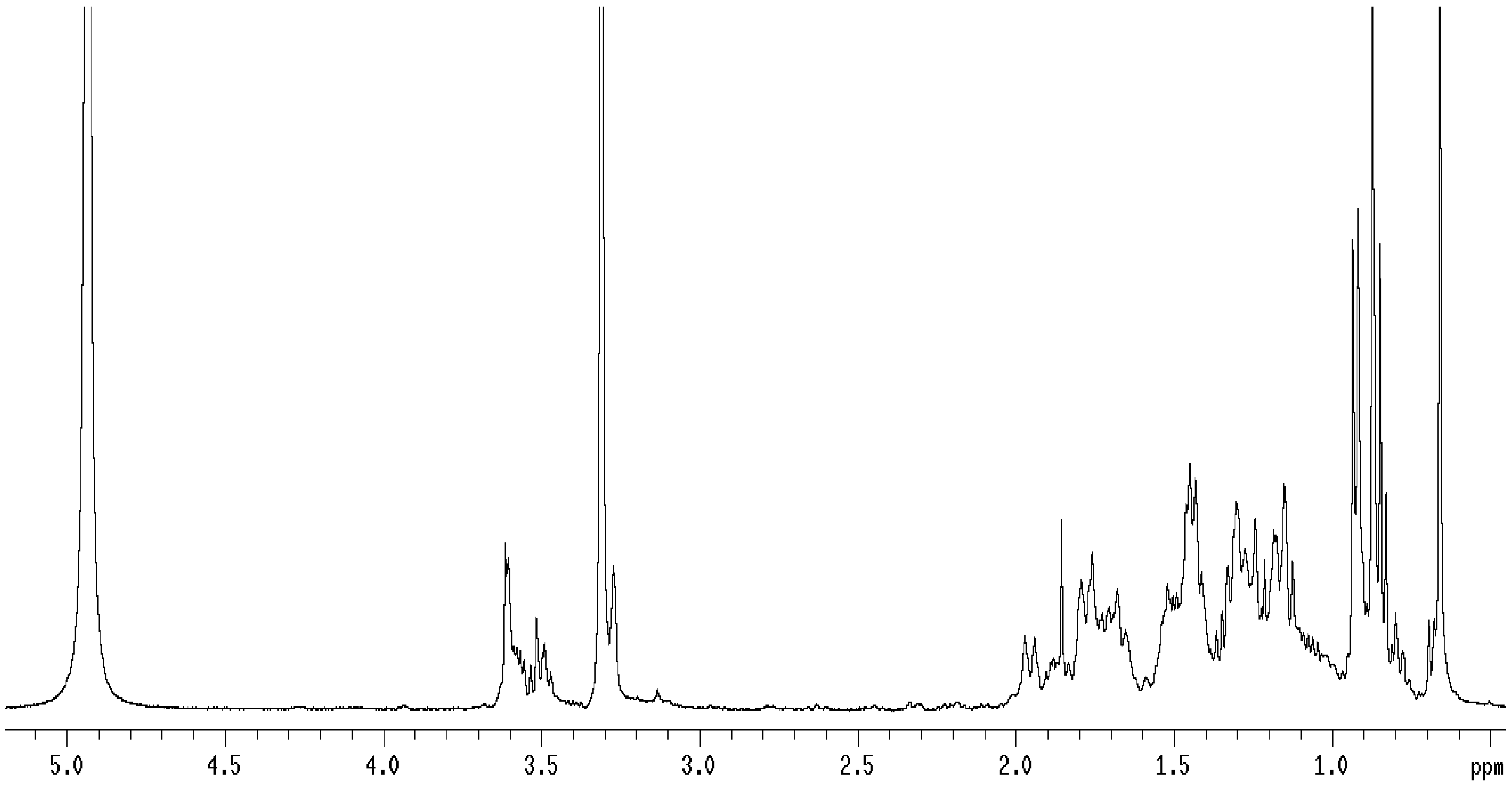

^{1}H NMR (400 MHz, CD_3OD) of compound **13**

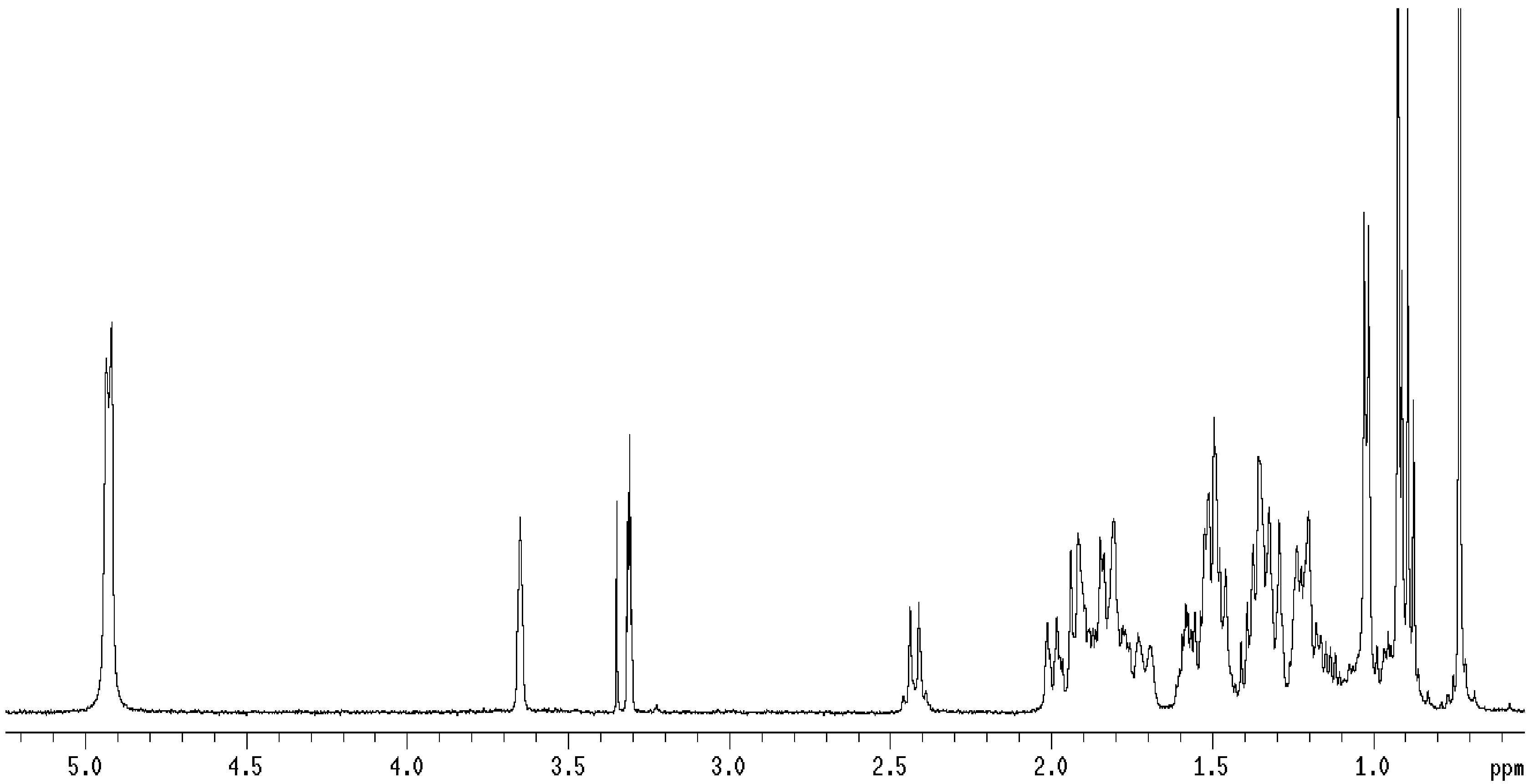

^{1}H NMR (400 MHz, CD_3OD) of compound **14**

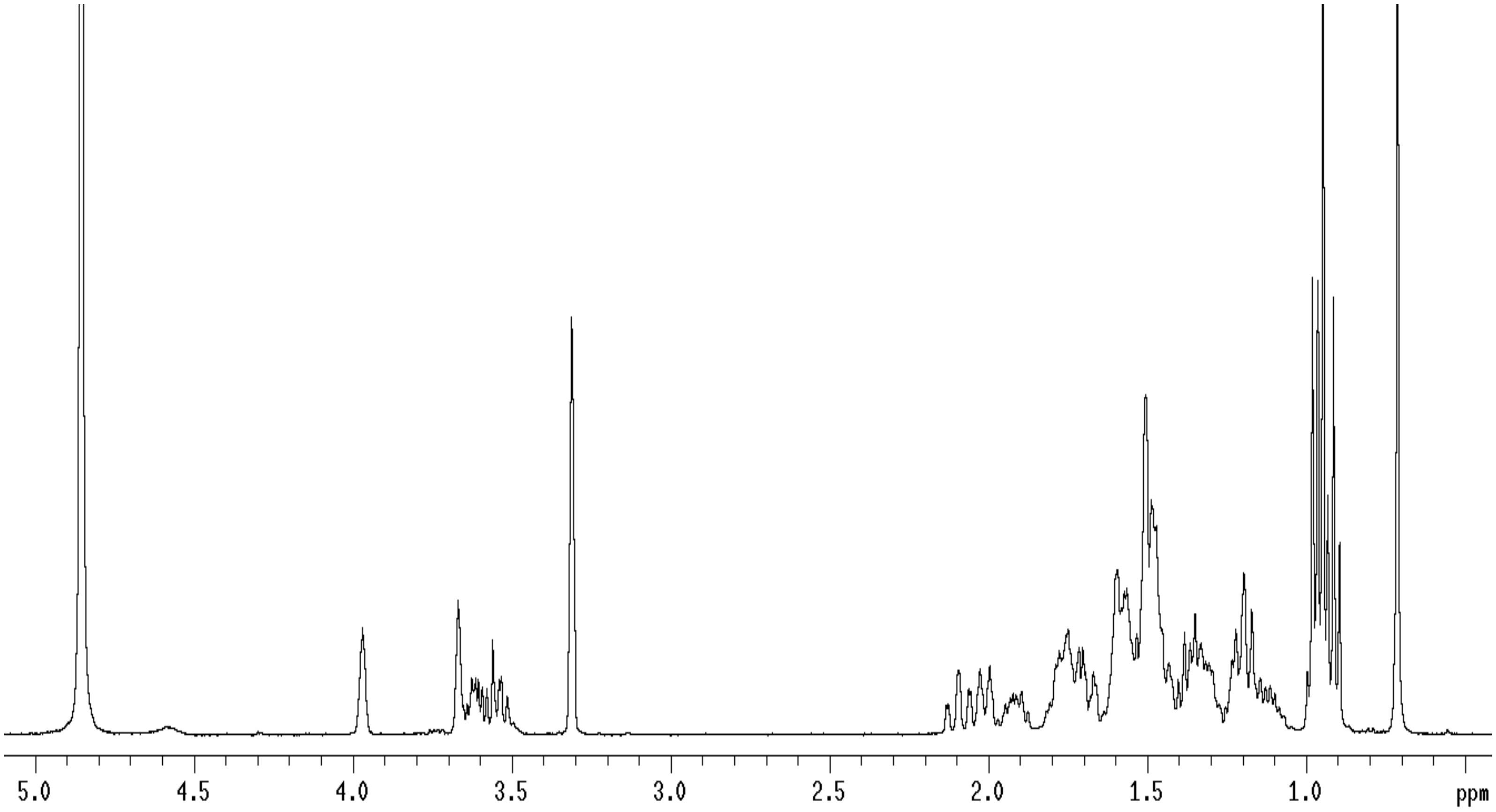

^{1}H NMR (400 MHz, CD_3OD) of compound **15**

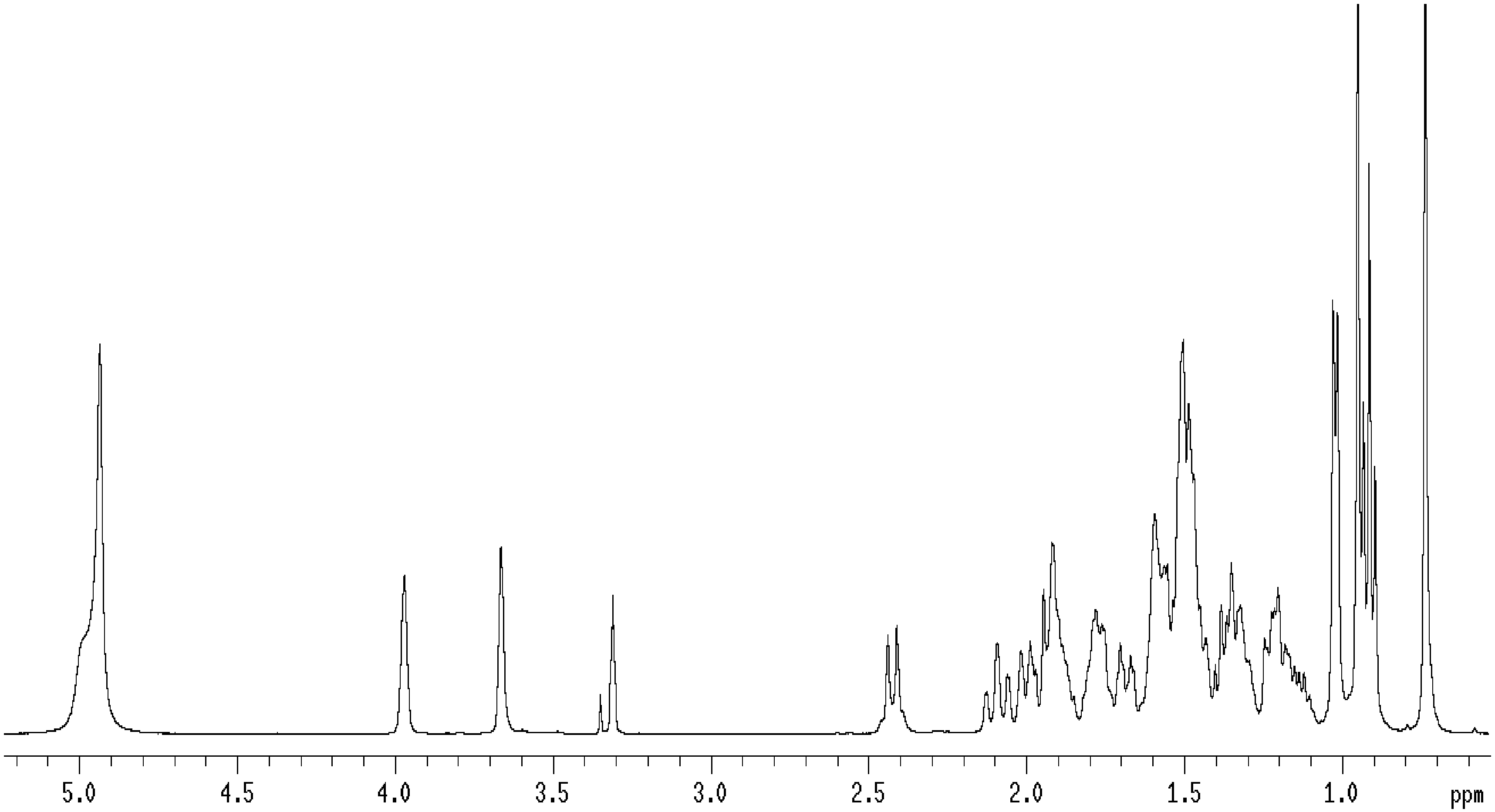

^{1}H NMR (400 MHz, CD_3OD) of compound **16**

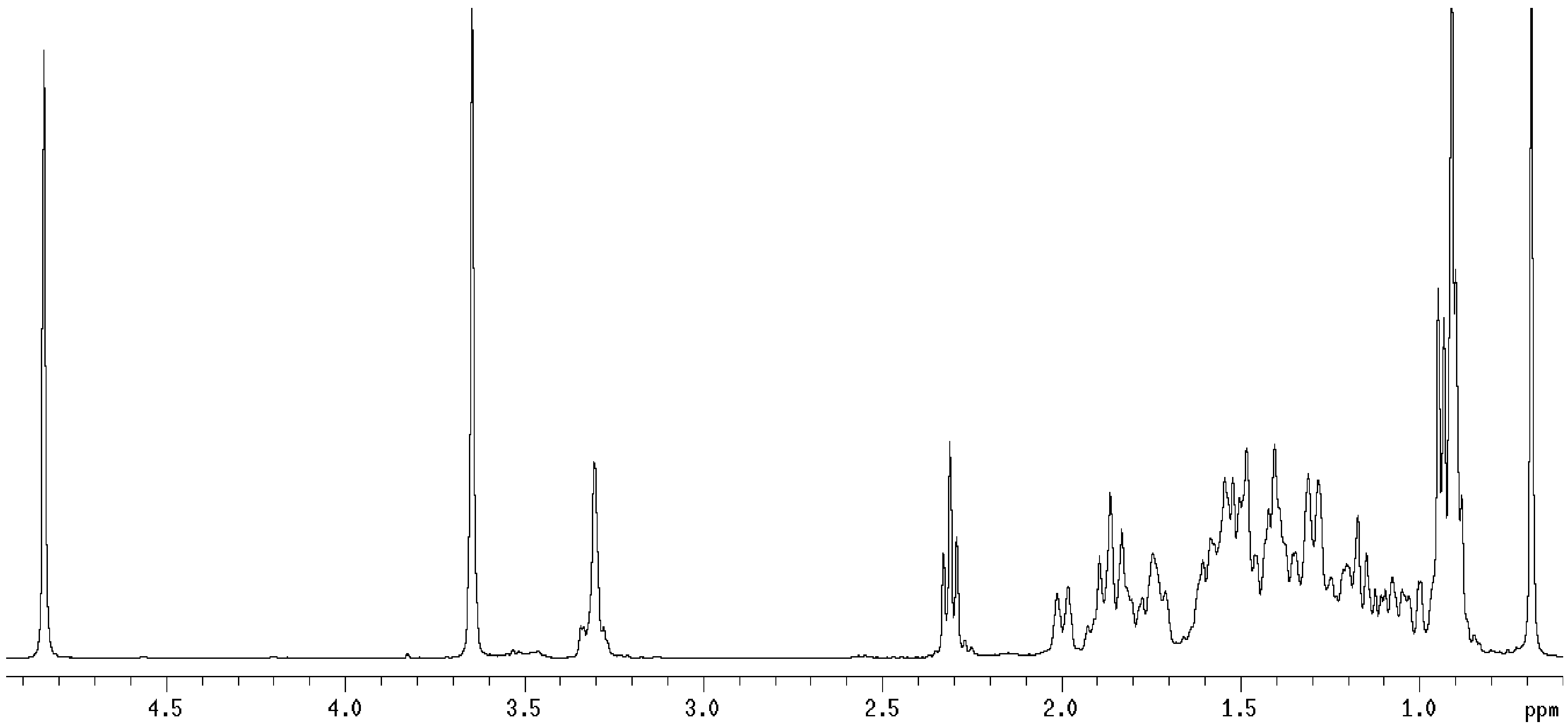

^{1}H NMR (400 MHz, CD_3OD) of compound **17**

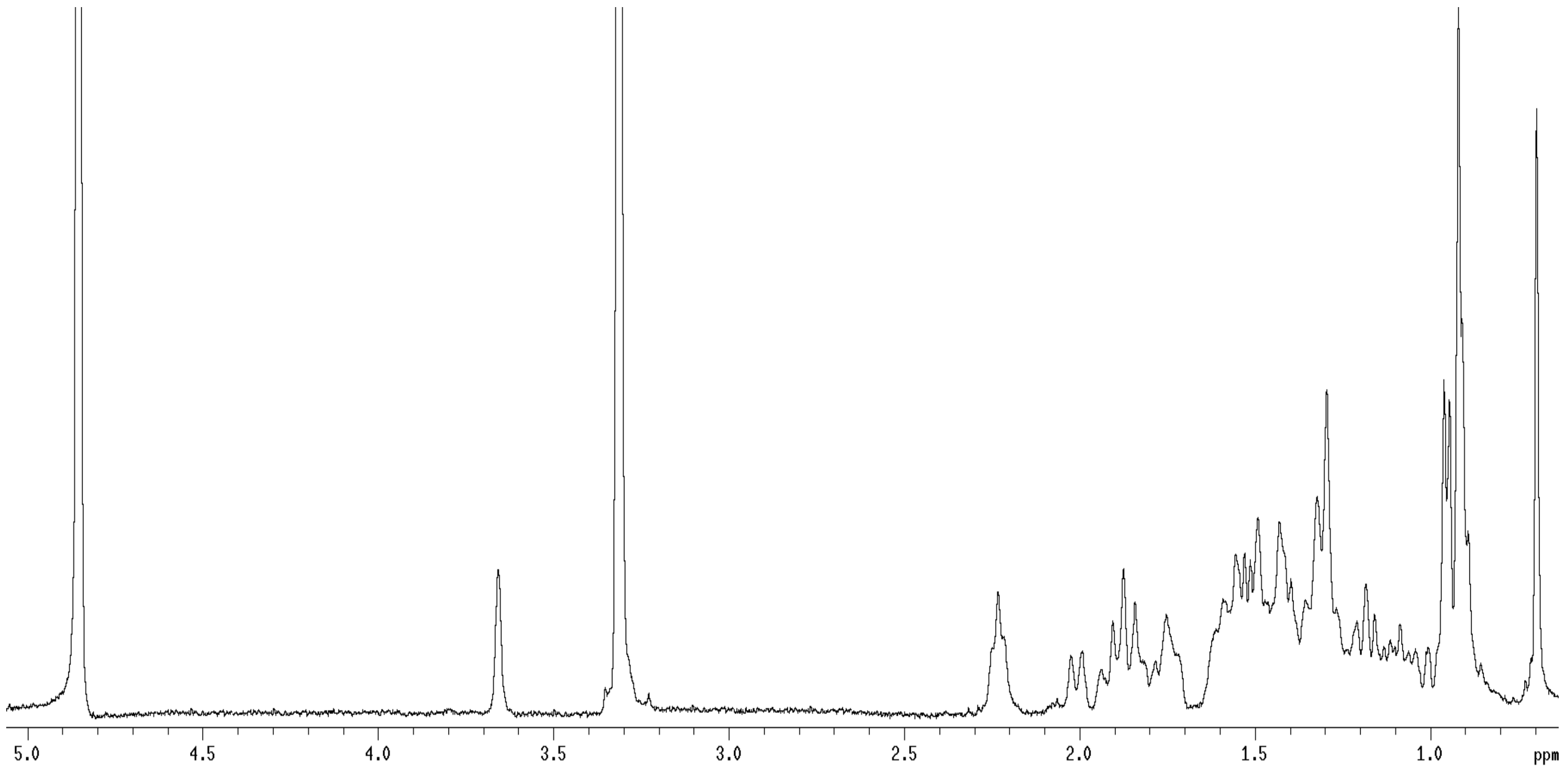

^{1}H NMR (400 MHz, CD_3OD) of compound **18**

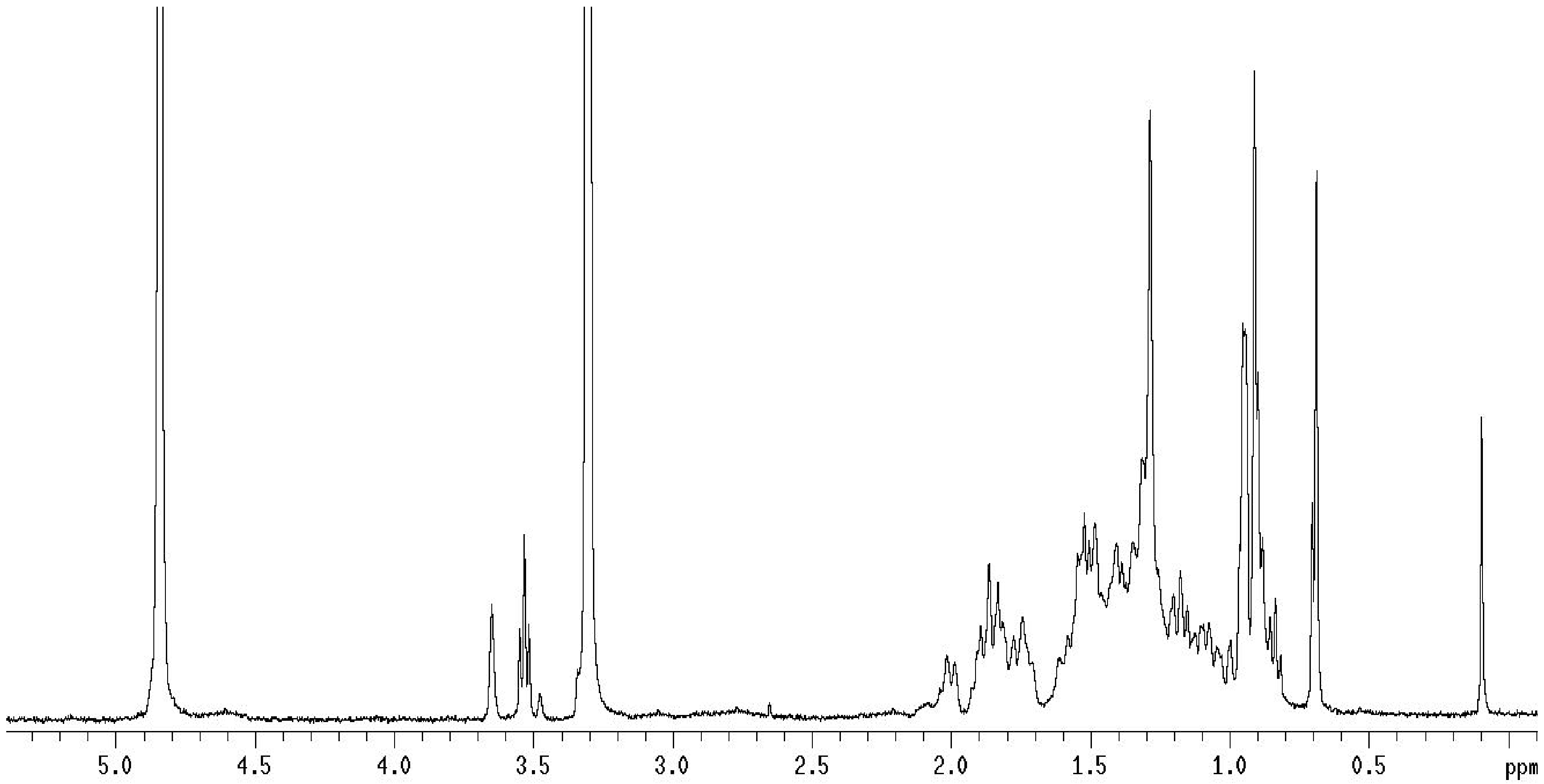

Supplement: Supplementary Information [file srep29320-s1.pdf]
